# The path from big data analytics capabilities to value in hospitals: a scoping review

**DOI:** 10.1186/s12913-021-07332-0

**Published:** 2022-01-31

**Authors:** Pierre-Yves Brossard, Etienne Minvielle, Claude Sicotte

**Affiliations:** 1 Arènes (CNRS UMR 6051), Institut du Management, Chaire Prospective en Santé, École des Hautes Études en Santé Publique, Rennes, France; 2 i3-Centre de Recherche en Gestion, Institut Interdisciplinaire de l’Innovation (UMR 9217), École polytechnique, Palaiseau, France; 3 Institut Gustave Roussy, Patient Pathway Department, Villejuif, France; 4 Department of Health Management, Evaluation and Policy, University of Montreal, Quebec, Canada

**Keywords:** Big data analytics, Hospitals, Value creation, Capabilities, Resource-based view

## Abstract

**Background:**

As the uptake of health information technologies increased, most healthcare organizations have become producers of big data. A growing number of hospitals are investing in the development of big data analytics (BDA) capabilities. If the promises associated with these capabilities are high, how hospitals create value from it remains unclear. The present study undertakes a scoping review of existing research on BDA use in hospitals to describe the path from BDA capabilities (BDAC) to value and its associated challenges.

**Methods:**

This scoping review was conducted following Arksey and O’Malley’s 5 stages framework. A systematic search strategy was adopted to identify relevant articles in Scopus and Web of Science. Data charting and extraction were performed following an analytical framework that builds on the resource-based view of the firm to describe the path from BDA capabilities to value in hospitals.

**Results:**

Of 1,478 articles identified, 94 were included. Most of them are experimental research (*n*=69) published in medical (*n*=66) or computer science journals (*n*=28). The main value targets associated with the use of BDA are improving the quality of decision-making (*n*=56) and driving innovation (*n*=52) which apply mainly to care (*n*=67) and administrative (*n*=48) activities. To reach these targets, hospitals need to adequately combine BDA capabilities and value creation mechanisms (VCM) to enable knowledge generation and drive its assimilation. Benefits are endpoints of the value creation process. They are expected in all articles but realized in a few instances only (*n*=19).

**Conclusions:**

This review confirms the value creation potential of BDA solutions in hospitals. It also shows the organizational challenges that prevent hospitals from generating actual benefits from BDAC-building efforts. The configuring of strategies, technologies and organizational capabilities underlying the development of value-creating BDA solutions should become a priority area for research, with focus on the mechanisms that can drive the alignment of BDA and organizational strategies, and the development of organizational capabilities to support knowledge generation and assimilation.

**Supplementary Information:**

The online version contains supplementary material available at 10.1186/s12913-021-07332-0.

## Background

There is a strong belief that health information technologies (HIT) are essential for improving the performance of health care organizations (HCOs) [1]. Convinced by the potential benefits – either clinical, operational or economic – of these new technologies, policy-makers and public agencies in Europe and North America have been committing significant financial resources to accelerate the HIT uptake in HCOs [2].

Today, most HCOs use HIT intensively, becoming *de facto* producers of large volumes of data in digital form. These digital data come from the different components of local health information systems (HIS), including electronic medical records (EMR) and can be either structured or unstructured [3]. These data can be labelled as big data. As first formalized by Laney [4], big data are “high-volume, high-velocity, and/or high-variety information assets that require new forms of processing to enable enhanced decision making, insight discovery, and process optimization”. This definition is also known as the 3Vs paradigm. The abundance of data confers importance to big data analytics (BDA) that encompass the technologies and techniques that support the processing of these 3Vs [5] to derive knowledge that can drive improvements in quality, security and efficiency of care delivery [6]. Today, the application of BDA is identified as a key success-factor in health care reforms or transformations [7].

In this environment, and as BDA technologies and techniques have become widely available, a growing number of hospitals invest in the development of BDA capabilities (BDAC). With these investments, hospitals hope to generate knowledge that will help them drive change in their strategies, practices and organizations and better cope with internal and external pressures [8]. Hospitals’ investments in BDA are increasing at a rapid pace and are focused on the acquisition and implementation of BDA technologies. These investments can prove risky and lead to important financial losses if they do not deliver on their promises, especially in an environment exposed to budgetary and organizational constraints like those hospitals are confronted with.

How the value creation potential of BDA is realized by hospitals remains unclear. Despite the fast growth pace of BDA in health care research [9], very few scholars have addressed value creation from BDA capabilities in hospitals. Most of the literature is focused on the development of these capabilities and the associated technological choices. The few references that cover value creation do so from a macro or sectorial perspective [10, 11], minimally addressing it at the single organization level.

## The research question

Our main research question is to understand how value is created from the use of BDA in hospitals. Answering this question requires to map the path from BDA capabilities to value, gathering evidence on the BDA capabilities that are leveraged, the mechanisms that mediate value creation, the value targets that are pursued and, eventually, the generated benefits. Exploring the path from BDA to value in a diverse set of hospital contexts can help understand challenges hospitals are facing when investing in BDA technologies.

To fill this gap in understanding, we decided to conduct a scoping review, in which we systematically searched, reviewed and analyzed the content of scholar articles covering BDA applications in hospital contexts to propose a comprehensive overview of research on BDA use in hospitals and map the components of the value creation process from BDA. We opted for a scoping rather than a systematic review because scoping reviews are exploratory in nature [12] and particularly relevant to provide a comprehensive overview of the diversity of approaches that characterize a broad topic like the one we study [13].

To better deal with the diversity of hospitals’ contexts, strategies, organizations and practices, we followed a descriptive analytical method [14] examining all studies in relation to a common analytical framework defining the components of the path from BDA to value. We developed this framework building on the resource-based view (RBV) of the firm [15–17],

By synthesizing the knowledge on how hospitals leverage the BDA capabilities they invest in, our ambition is to help health authorities and hospital managers gain a better understanding of how value is created from big data to better steer their BDA strategies and projects.

## An analytical framework for analyzing the literature

To systematically explore the literature on BDA applications in hospitals, we developed an analytical framework building on the resource-based view (RBV) of the firm [15–17]. RBV is the most frequently used management theory in big data research [18]. According to RBV, resources owned by firms are inputs that cannot generate value by themselves [16, 17, 19]. Competitive advantage derives from the strategic bundling of valuable resources into capabilities. These capabilities, which are firm-specific constructs and difficult to imitate, will support value creation and competitive advantage.

Hospital managers are currently investing in data assets, analytics technologies and techniques, and human skills to develop the infrastructure on which to develop BDA capabilities [20]. These capabilities are essential to manage the volume, variety and velocity of available data and to generate valuable results from their analysis [21]. A challenge for hospital managers is to select which bundling strategies to operate in order to develop the capabilities that will deliver valuable outputs [22, 23] and justify the investments made. Based on the work by Wang and Hajli [24], the main BDA capabilities to support value creation in healthcare are: a) traceability, b) interoperability b) analytical capability, c) predictive capability, d) decision support capability.

According to the RBV theory, once developed, these capabilities can support multiple value-creating needs for hospitals. This study expands the RBV to question how BDA capabilities support value creation and for what benefits. The exploration of the value creation process from BDA capabilities starts with the mechanisms which mediate the link between BDAC and value. These value creation mechanisms (VCM) are sources of value but not value themselves as it is sometimes mistaken in the literature [10, 25]. It is important to reposition VCMs in the debate for what they are: Enablers of value creation and organizational transformation. Building on existing literature [25], we identified and adapted to the hospital context five value creation mechanisms: creating process and outcomes transparency, enabling discovery and experimentation, supporting customization of actions through segmentation, enabling optimization through prediction, and enabling real time monitoring of activities and outcomes [25, 26].

These mechanisms are expected to enhance hospital professionals’ abilities to drive change in their practices and organizations. Looking into the selected literature, the review describes what are the value targets set by hospital professionals.

Eventually, reaching these targets can generate benefits for hospitals. We distinguished expected and measured benefits based on a classification adapted from the framework developed by Shang and Seddon [27] which defines 4 types of benefits: operational, organizational, managerial and strategic.

**Fig. 1:**
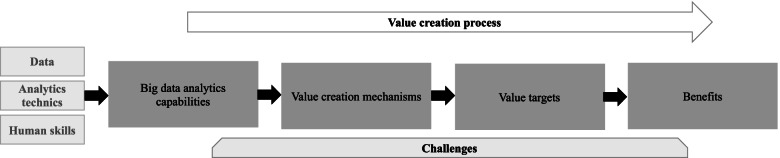
Analytical framework

By applying the framework summarized in Fig. 1 to review the literature on BDA applications in hospitals, we aim at shedding a light into the black box between BDAC and value. If RBV asserts that capabilities are essential to support value creation, a better understanding of how these capabilities are realized is needed.

## Methods

We conducted our scoping review following Arksey and O’Malley’s five stages framework [12] and applied the Preferred Reporting Items for Systematic Reviews and Meta-Analyses for Scoping Reviews (PRISMA-ScR) Checklist for reporting [28] (Supplementary Table 1 in Appendix).

**Table 1:** Inclusion and exclusion criteria

Criterion	Inclusion	Exclusion
Time period	2014 to 2019 (95% total sample)	Articles published before or after this time period
Language	English	Other publication languages
Document type	Articles and reviews	Other type of documents
Study focus	Research involving the application of BDA	Articles focused on technologies with no application
Study setting	Application of BDA in a hospital setting Application of BDA on hospital activities (care, research, education, operations)	Other care settings (primary care, non-hospital-specific activities)
Literature focus	Explicit mention of value targets or benefits of BDA applications	No perspectives on value creation

**Stage 1** relates to the identification of the initial research questions. As previously mentioned, our main research question is to understand how value is created from BDA capabilities in hospitals. We will answer it by investigating a set of secondary questions related to the different components of our analytical framework:

- What are the BDAC leveraged by hospitals to create value?
- What are the mechanisms that mediate the link between capabilities and value?
- What are the value targets and benefits?
- What are the challenges associated with value creation from BDAC?

**Stage 2** consists in identifying the relevant articles for the review. We adopted a systematic search strategy and applied it to two of the largest citation databases: SCOPUS and Web of Science [29]. Search terms were chosen to capture a literature that relates to the application of BDA in hospitals. We searched for a combination of two keywords. The first keyword was “hospital” and its derivatives (hospital OR hospitals OR "health org*" OR "healthcare org*" OR "health care org*" OR "health cent*" OR "healthcare cent*" OR "health care cent*" OR "medical cent*" OR "medical inst*") to narrow down the scope of the results to the hospital setting. The second keyword related to the technologies and techniques we were interested in. We settled on two terms: “big data” and “data analytics”. These terms were expected to capture most researches on big data and their associated techniques, including the *en vogue* terms of data mining and machine learning.

Articles were searched initially in September 2020 and updated in November 2020 and January 2021. We collected 1,478 references with our initial searches once duplicates identified and deleted. We then added a set of exclusion criteria (Table 1) to reduce the number of candidate articles to be analyzed. It is important to note that to capture the diversity of hospitals’ approaches to value creation from BDA and examine if geographic and political contexts have any influence, we opted not to restrict country of authorship in our search.

**Fig. 2:**
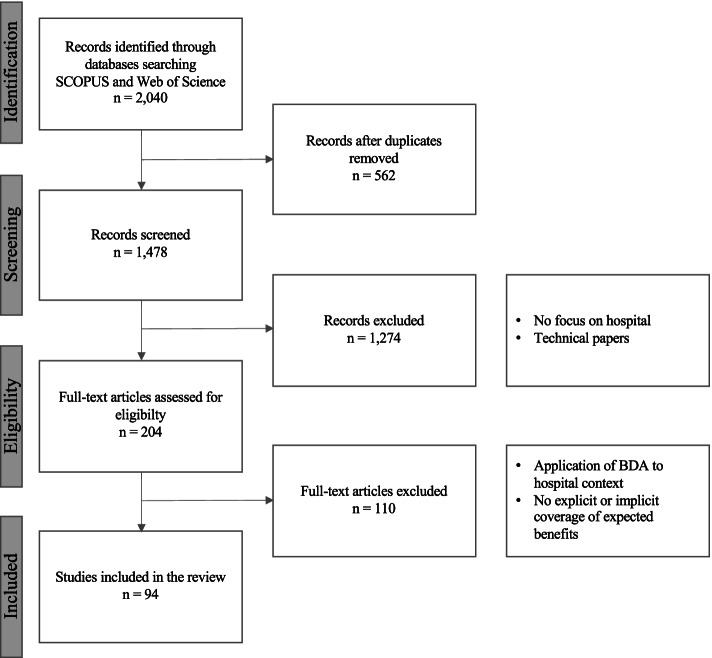
PRISMA flowchart: Search and selection process

**Stage 3** involves article selection. The members of the research team iterated all along the process to discuss and refine the search strategy. The first author conducted the initial screening on titles and abstracts which led to the exclusion of 1,274 articles. The same author then performed a full-text review of the 204 remaining articles which led to the exclusion another set of 110 articles that did not meet inclusion criteria. At the end of the search and selection process, 94 articles were considered as relevant for analysis (Figure 2).

**Stage 4** involves data charting and data extraction. We designed coding grid on Microsoft Excel which was structured around the variables of our analytical framework. These variables were declined in categories taken from existing literature sources [24–27]. We then followed a descriptive-analytical method [14], with the coding consisting in extracting text fragments and associating them with the pre-defined categories. The grid was tested on 10 articles. Minor adjustments were made to the grid before starting the analysis of the full sample. The first author performed this analysis with advice from the other authors when needed. At the end of the coding process, categories were adjusted and subcategories introduced.

**Stage 5** consists in reporting the results. We started with describing the included studies before segmenting our presentation of results into thematic analysis for each variable of the analytical framework.

## Results

Our findings are presented in the next sections. We first describe the study characteristics (1) before exploring more thoroughly the value creation process from BDAC (2) and its associated challenges (3).

### Study characteristics

Big data analytics emerged in the healthcare research literature in 2008 with the volume of articles exploding in 2014 [9]. With 95% of articles published between 2014 and 2019 our initial search results (1,478 articles) are aligned with this trend. This dynamic is also confirmed in our final dataset with 57 of 94 articles being published in 2018 and 2019.

Most articles are experimental (*n*=69). They are proofs of concepts of different BDA functionalities and techniques to deal with hospital-specific clinical or administrative challenges. They cover both theoretical and practical contributions. Twenty two articles are case studies investigating the realization of BDA capabilities in hospitals, or groups of hospitals, that position themselves as early-adopters of these new technologies [30].

Using the All-Science Journal Classification (ASJC), we categorized the selected articles by research fields. Medicine and computer science are associated respectively with 66 and 28 articles. Other fields are engineering (*n*=9), nursing (*n*=6), decision science (*n*=6). Business and management are marginal, accounting for only 4 articles.

The summary of study articles and the description of the path-to-value from BDAC are presented in Supplementary Tables 2 and 3 in Appendix.

### Value creation process from BDA capabilities

#### Big data analytics capabilities

The 5 types of capabilities we classified are associated with the 3 main steps of the BDA process [31]: data acquisition, data analysis and data interpretation.

##### Data acquisition (*n*=54)

Traceability and interoperability are two types of BDAC associated with this first step of the BDA process. They are respectively mentioned in 43 and 28 articles.

Traceability is the ability to track data from different complex data sources and/or HIT components. For instance, BDA techniques can help identify and track surgical complications [32], drug-drug interactions [33] or opioid use among hospitalized patients [34] from different sources to generate new datasets for further analysis.

Interoperability is essential to aggregate data from multiple sources and locations and to integrate them into a single data structure [6]. This capability is essential to support the constitution of databases from different sources [35] and the linking of databases from different parts of the HIS [36, 37].

##### Data analysis (*n*=94)

Analytical capability and predictive capabilities are both focused on data analysis.

Analytical capability is the most frequently mentioned capability in our research (*n*=58). It is the ability to use descriptive data analytics techniques such as data mining, text mining and statistics to generate new knowledge. For example, natural language processing techniques can help automate the extraction of disease-specific risk factors from unstructured data [38] or explore patients’ verbal complaints to identify text patterns that can be further analyzed to be associated with specific conditions [39]. The use of advanced statistics enables to describe population characteristics [40], characterize the features of a condition [41] or identify correlation between two dimensions such as staffing level and clinical outcomes [42].

Predictive capability is mentioned in 50 articles. Most applications of predictive analytics consist in leveraging machine learning (ML) techniques (*n*=39) to identify predictors of diseases or adverse events. For example, the application of ML on claims can help identify the determinants of in-hospital mortality [43]. It can also enable to risk stratification by identifying patient groups using ML clustering techniques. Finally, few articles [17], mostly in administrative settings, go further with quantified forecasts of outcomes with recommendations as demonstrated by the use of ML to support the development of a real-time prediction of intra-surgical remaining time [44].

##### Interpretation

Decision support capability appears in 44 articles. It relies significantly on data visualization (*n*=27) and often complements analytical and predictive capabilities (respectively in 33 and 18 articles). It is the ability to effectively generate outputs in a format that is actionable. For example, the use of dashboards on activities and movements of care resources can help guide decision on resource allocation [45], the development of visual timelines on patient medication can help better focus on when and in which context adverse events occur [46] or the use of continuous display of information from cardiorespiratory monitoring in the ICU can help better anticipate septic shock [47].

**Fig. 3:**
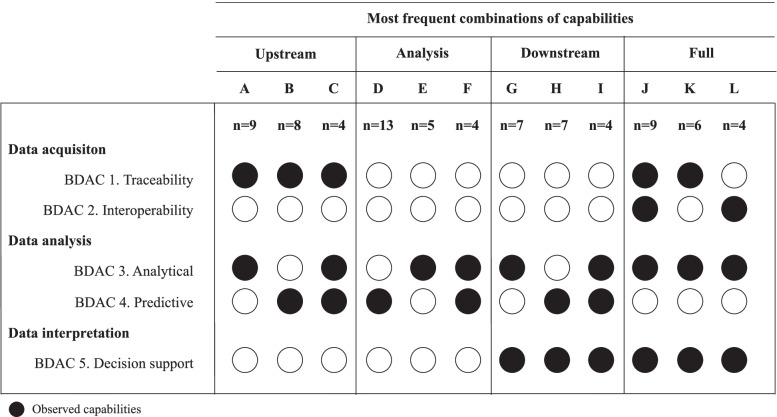
Capabilities combinations

To acquire, analyze and interpret data, the literature indicates that hospitals do not rely on a single capability but on combinations. 28 articles present combinations of capabilities that are focused on the upstream part of the big data analytics process (data acquisition and data analysis), 18 on the downstream part (data analysis and data interpretation) and 26 on the full process (data acquisition, data analysis and data interpretation). In Figure 3, we present the most frequent combinations of capabilities and their frequency.

#### Value creation mechanisms

As value creation mechanisms were not explicitly identified in the selected articles, we deducted them following existing classifications developed by Manyika [26].

Creating process and outcomes transparency (VCM1) is identified in 42 articles. It is a fundamental source of value from BDA for hospitals which can gain complete and consistent visibility on processes and outcomes. Process transparency (*n*=17) enables change in practices and organizations. For example, the adoption of a comprehensive data analytics platform enables clinical and administrative staffs to better identify variations in clinical outcomes, supplies, labor and costs and support the redefinition of practices and organizations for operational gains [48]. Outcomes transparency (*n*=25) can support quality improvement initiatives. For example, BDA can facilitate the development of risk-adjusted quality assessment models that can be applied to benchmark performance between services, institutions or healthcare systems and act as a driver of change [32].

Enabling discovery and experimentation (VCM2) is the most common VCM in our research (*n*=92). BDA enables hospitals to explore routinely collected data at different levels of their organization to unearth valuable insights or knowledge patterns. It enables staffs to transform data into actionable wisdom [49] that can be leveraged at different levels of their organizations. From a clinical perspective, Moss [50] demonstrates how advanced statistical tools enable the discovery of signatures of potentially catastrophic illnesses to guide care optimization. From a managerial perspective, data mining techniques can help discover patterns on the relationships between staffing level and clinical outcomes on which models for acute staffing can be developed [42].

Supporting customization of actions through segmentation (VCM3) is mentioned in 46 articles. Classification, clustering and other techniques enable differentiating patient populations to drive case customization. Machine learning can outperform existing clinical decision rules to classify patients based on their risk profiles [51] or enable the automation of resource-intensive classification activities such as triage in the emergency department [52, 53]. Segmentation can drive efforts to customize practices and organizations and improve both clinical and operational outcomes.

Enabling optimization through prediction can mediate value creation (VCM4). The ability to determine the best path from the use of BDA appeared in 19 articles. Data-driven determination of probabilistic outcomes paves the way for prescriptive analytics whose ambition is to prescribe action plans to increase the likelihood of the occurrence of a desired outcome [6]. For example, by outperforming human judgement in the prediction of patient flow, statistical models can contribute to recommend optimized resource allocation strategies to avoid wastage and unnecessary spending [54].

Enabling real time monitoring of activities and outcomes (VCM5) is mentioned in 25 articles. Monitoring patients or activities come with a heavy burden of information processing that is time consuming for resource-restrained hospitals. BDA applications not only unload staff members from these activities, but offer the opportunity to increase the volume and the depth of data sources to monitor. Activities which would normally be beyond human capacity such as real-time monitoring of drug-drug interactions [33] or monitoring health status of neonatal patients from devices-generated data [55] are made possible by BDA applications to enable responsiveness.

It is interesting to note that these five mechanisms are different in nature. With transparency, value comes with the availability. With discovery, it comes from the content. With segmentation, prediction and monitoring, the value comes from the format.

In many instances, these value creation mechanisms need to be combined to put knowledge into actions. Analyzing the combinations confirms discovery as being the cornerstone of value creation from BDAC as it appears in the three most commonly observed combinations a) VCM2-VCM3 [46], b) VCM1-VCM2 (*n*=41) and c) VCM2-VCM5 [23]. These combination are associated with different logics. The association of discovery and transparency is focused on knowledge generation. It enables hospitals to increase their knowledge stock that can later be transferred to practice and decision-making settings. It is an exploratory approach to value creation from BDAC with the ambition being to find change opportunities and define targets. The combinations of discovery and segmentation or monitoring are focused on knowledge assimilation. The objective is to make knowledge applicable to drive changes in practices and organizations. It is an operational approach to value creation from BDAC. The targets to reach are known and BDA is leveraged to support practical changes required to reach them.

#### Value targets

BDA is a versatile technology that can apply to hospitals’ main activity domains which are care (*n*=67), administration (*n*=48) and research (*n*=8). In each of these domains, a variety of value targets can be set and reached. These targets differ in nature and serve a wide range of objectives. We identify 3 targets: improving quality of decision-making (VT1), driving innovation (VT2) and improving process performance (VT3).

Improving the quality of decision-making (VT1) is the most frequent value target pursued in the literature (*n*=62). The ability to generate and disseminate knowledge from available data coupled with incentives to act on these data or developing tools to augment human capability can enable improvement of decision-making.

In care, BDA applications can facilitate disease diagnosis (*n*=12) and risk detection (*n*=22) by accelerating the decision-making process or improving its precision. For example, BDA can enable the development of a personalized diagnostic model [56] or support the design of rapid diagnosis at point of care [57]. BDA techniques can also enable the identification of patients at risks of complications after surgery [43] or at risks of readmission after inpatient stays [58].

By enabling outcomes and process transparency, BDA can support administrative teams in assessing hospital activities (*n*=20). It can enable managers to gain visibility on the quality of care [32], assess performance of new organizations [59] and engage in consensus-building with clinical teams to drive operational changes [48]. BDA can also contribute to improving decision-making on resource allocation (*n*=14). Predictive analytics solutions can support optimization of hospital wards usage and management [45] or predict patient volumes to adapt staffing levels [60] or optimize safety stock of nurses staffing [61].

Regarding research activities, BDA can facilitate hypothesis-setting (*n*=2) with BDA tools enabling researcher in preliminary investigation to test the relevance of their research question or identify new research opportunities [62, 63].

##### The second value target is driving innovation (VT2)

It appears in 54 articles. By analyzing new insights, hospitals' clinical, administrative and research staffs can better identify opportunities for innovation that will drive changes in their practices and organizations. In care, the two main types of innovation pursued are precision medicine, which consists in personalizing care for better outcomes (*n*=27), and preventative medicine (*n*=22), which consists in changing the course of actions to avoid the occurrence of adverse events. BDA can support the emergence of precision medicine by enabling physicians to identify patients who will benefit more from specific therapies [64] or to predict prognosis to adapt the course of treatment [41]. BDA can create the conditions for innovative approach to risk management in care by enabling the identification of patients at risks of surgical complications ahead of intervention [65] or at risks of pressure ulcer during inpatient stays [66].

For managers, BDA can be used as a tool to adapt strategies (*n*=10). It can support the design of new payment models for patient subgroups by predicting costs of care [67]. The interest of these models is two-fold: secure operational margin and cost control and revenue level, and impact competitors that will need to adapt to new delivery and financing schemes.

For research teams (*n*=2), BDA can enable the development of innovative tools to support the emergence of new clinical trial designs and facilitate the identification of patient selection for recruitment [68], contributing to reinforcing the competitiveness of research activities.

##### The last value target we identified is performance improvement (VT3)

Enabling process transparency and facilitating continuous monitoring of activities can contribute to process improvement all along the hospital value chains (*n*=36).

Optimization of patient flow in care units is a common value target in care (*n*=25) as BDA can support patient waiting-time reduction by optimizing scheduling [45], or improve inpatient capacity usage through continuous prediction of inpatients length of stays [67].

BDA can support the optimization of hospital operations (*n*=7). Procurement [54], waste-management [69] or equipment management [70] can benefit from the use of BDA techniques.

This also applies to research (*n*=6), where the use of BDA has considerably facilitated patient screening and selection to perform retrospective studies [68].

BDA applications and their value targets are presented in Table 2.

**Table 2:** Distribution by value targets

Value targets	N	References	Description
**VT1 – Decision making**	**62**		
Care – Diagnostic	12	Hu et al. (2018) [71]	Develop models based on machine learning to aid the diagnosis of hyperlemia at point of care.
Care – Risk detection	22	Genevès et al. (2018) [72]	Use machine learning on prescription data to detect, on the day of hospital admissions, patients at risks of developing complications during their hospital stay.
Admin. – Assessing hospital activities	20	Mahajan et al. (2019) [73]	Develop a data-driven methodology for decision-making supported by the use of quarterly strategic analytics for improvement and learning (SAIL) reports to visualize data, study trends and provide actionable recommendations.
Admin. – Resource allocation	14	McNair (2015) [61]	Use statistical model to forecast the optimal safety level of nurse staffing in intensive care units.
Research – Hypothesis setting	2	Hendricks (2019) [62]	Use process mining to explore available hospital logs and identify areas in clinical operations to further investigate.
**VT2 – Innovation**	**54**		
Care – Precision medicine	27	An et al. (2018) [74]	Develop algorithms using machine learning methods to predict drug-resistant epilepsy in order to ensure these patients receive specific care and interventions following their diagnosis.
Care – Preventative medicine	22	Zolbanin and Delen (2018) [75]	Propose new data processing approaches to predict preventable readmissions for patient with chronic diseases and prescribe the best course of actions for each patient at discharge to prevent readmission.
Admin. – Adapt strategies	10	Navarro et al. (2018) [67]	Develop a machine learning algorithm using perioperative data to predict length of stay and inpatients costs after primary total knee arthroplasty and propose a patient-specific payment model better reflecting patient complexity.
Research – New research tools	2	Johnson et al. (2016) [63]	Develop a dynamic simulation tool suitable for data visualization of both human-designed and data-driven process which can be used for “what if” analysis and used to deep-dive on big data.
**VT3 – Performance**	**36**		
Care – Patient flow	25	Krämer et al. (2019) [76]	Use supervised machine learning techniques to train a model to classify inpatient admissions as either emergency or elective care to reduce the number of hospitals admissions from the emergency department.
Admin. – Operations management	7	Guan et al. (2017) [54]	Use statistical model to investigate platelet usage patterns and better forecast future demand to reduce wastage.
Research – Research performance	6	Karanastasis et al. [68]	Develop a platform with tools and services necessary to explore big data in clinical research to improve the efficiency of clinical trials design and the effectiveness and speed of subject recruitment.

#### Benefits

Hospital professionals associate benefit expectations with the definition and pursuit of value targets. These benefits are detailed in the Supplementary Table 4 in Appendix. The benefits that are the most frequently expected are operational (*n*=83). BDA is expected to help improve the quality of clinical decisions (*n*=34) [39, 45, 57, 77] and outcomes (*n*=27) [37, 53, 54, 58, 61, 78]. It is also expected to enable cost-reduction (*n*=38) by reducing unnecessary care [41, 56, 64, 79–81] and admissions [58, 82, 83]; productivity gains (*n*=26) by optimizing resource usage in care and administrative units [32, 84, 85]; or service improvement (*n*=27) by providing healthcare professionals new operational tools to support their practices [44, 82]. 

Organizational benefits are expected from BDA in 62 articles. These changes are observed in individual practices (it may be diagnosis, prescription, orientation) [34, 86] with BDA acting as an enabler of learning and skills development (*n*=21). It can also support changes in work patterns to drive new care organizations (coordination, pathways, access to technical resources) (*n*=41) [67, 76, 87]. Finally, BDA can enable better communication and collaboration to change ways of working (collectively, cross-functionally) (*n*=17) [63, 75].

Finally, the contribution of BDA to managerial and strategic benefits are expected in respectively 52 and 26 articles. The optimization of resource management [44, 54, 69, 88] is the main managerial impact considered in the reviewed articles (*n*=31), followed by performance improvement (*n*=22) [30, 48, 89] and improved decision-making (*n*=18) [90–92].

As for strategic benefits, the main feature is differentiate hospitals from other healthcare organizations (*n*=12) as mentioned by Ramkumar [79], Karanastasis [68] and McNair [61]. BDA can also be leveraged to support business innovation (*n*=9), which translates into the capacity to offer new value propositions to internal or external stakeholders, by either financing [79, 93], or way of working [30]. Improved strategic positioning (*n*=7) is another feature. It can take the form of increased attractiveness of care resources [30, 61] or reinforced influence over internal and external stakeholders [66, 89, 94].

If expectations are numerous, measured benefits are scarce. Only 19 articles quantify or describe benefits of BDA applications. This observation is not surprising given the experimental nature of the literature reviewed. Most of the measured benefits are operational (*n*=13) with cost-reduction being monitored in 6 articles [30, 48, 53, 59, 79, 82], quality improvements [47, 79, 95, 96] and service improvement in 4 articles each [79, 85, 97, 98] and productivity gains in 2 articles [95, 99].

Organizational benefits are assessed in 6 articles with focus on changing work patterns (*n*=4) [63, 90, 96, 99] and improving communication and collaboration (*n*=3) [79, 86, 96].

Finally, managerial and strategic benefits appeared, respectively, in 6 and 4 articles. The limited focus on managerial and strategic impact is coherent with the nature of the literature reviewed. Most articles in our datasets discuss BDA applications at a micro level, hence setting their focus on micro benefits that translate into operational and organizational perspectives.

#### Challenges to value creation

Many challenges hinder the value creation process described in the previous sec tions. These challenges impact the generation of valuable knowledge (*n*=79), the transformation of knowledge into actions (*n*=42), and eventually the ability to the develop a BDA strategy (*n*=21).

##### Generation of valuable knowledge

Developing BDAC to generate valuable knowledge require to properly orchestrate data, technologies and human resources.

Data-related challenges are the main source of concerns in selected articles. As obvious as it may sound, the generation of valuable knowledge from BDAC start with data access and quality.

Access challenge to data sources is mentioned in 48 articles. Access issues are observed between institutions [54, 58], different units in the same organizations [83] and different IT components [52]. They can be caused by organizational complexity [99], limited incentives to share data [99] or a lack of shared standards [42].

Data quality is mentioned 33 times with veracity of data being in question. Veracity is defined as “uncertainty due to data inconsistency and incompleteness, ambiguities, latency, deception, model approximations” [100]. Inconsistencies are caused by data collection systems and processes that are different from one site to the other [54], inconsistent use of medical terminology [49], and variance in coding practices between sites or within teams [50, 101]. Most datasets are incomplete [102] with missing variables [32, 103, 104] and values [105, 106] or underreporting of certain conditions [66].

While most BDA methods can be generalized, results often cannot [44, 56] because of access and quality issues. Hospitals lack tools and training to effectively and responsibly assess BD quality, preventing the effective use of BDA capabilities [107].

Technology-related challenges also impact hospitals’ ability to generate valuable knowledge (*n*=49). Acquiring and implementing BDA technological infrastructures is new and complex to many hospitals. It requires building on reliable preexisting IT infrastructure [51] and making the right combination of frameworks and software [82]. To make these choices, hospitals need to develop teams with sufficient BDA skills and talents to capitalize on the promise of big data. The availability of, or the ability to acquire, relevant BDA skills is a major challenge to the BDAC building process with most hospitals encountering difficulties to attract rare resources such as data engineers, data scientists and biostatisticians [79].

Capitalizing on BDA infrastructures, also requires hospitals to develop multi-disciplinary teams [38] associating technological experts and clinicians. Engaging with clinical teams in BDA projects often proves challenging as finding the right level of engagement to build trust [37] is made difficult by the heavy workload required [50] and the limited time these professionals can dedicate to such initiatives [94].

Hospital teams are also dealing with methodological challenges (*n*=16). When hospitals manage to create knowledge, the value of this knowledge is put in doubt. The validity of models is often in question and needs to be demonstrated before generalization [79]. Knowledge generated from the use of retrospective data would need to be confirmed with prospective studies [47, 76]. Experiments performed on a single site expose models and results to the influence of the context of experiment and introduce variability and hinder their reproducibility [99]. Solutions should be tested on multiple datasets to ensure a s imilar level of performance can be achieved at independent sites [108] which is a prerequisite to portability, adaptability and economic viability of BDA models [43, 84].

Finally, the lack of talents, or inability to engage them in data analysis activities restricts the ability to create valuable insights (*n*=17). Many organizations are facing difficulties to attract data scientists [30] and, as a consequence, lack the necessary skills to properly analyze and exploit data [81]. Developing BDA solutions to generate knowledge requires bringing clinical and technical expertise together. Setting up these multidisciplinary teams is complex [48] as it is often difficult to engage clinical experts in these activities [37] because that would result in a heavy workload for professionals who already have limited availability [50, 94].

##### Transforming knowledge into actions

Knowledge generation from BDA can trigger in-depth changes that can eventually contribute to the reconfiguration of hospitals' organizational capabilities [30]. Despite the strong technological advances in leading hospitals, there still is a considerable gap between BDA expectations in the healthcare field, and the benefits actually realized on the ground [109]. 

This gap can be explained by the complexity of BDA solutions (*n*=19). Developing BDA solutions expose hospitals to a set of organizational challenges among which is the ability to produce intensive cross-organization efforts to develop and implement BDA projects [68]. The complexity of these projects requires time and budget [63]. It is a long-term process that goes beyond the initial phase of knowledge generation [95]. It requires change management efforts with the conception of integrated package to drive change efficiently [82].

For hospitals investing in BDA, acceptance of BDA solutions is a major challenge (*n*=33) as one of the main risks is to see professionals walk away from these new technologies. Discontent starts with the perception of BD as a burden, with data collection being often considered as a tedious task [32]. The results generated often lack interpretability [41] with a growing number of algorithms being designed as black boxes [97, 101]. Lack of transparency negatively impacts trust of practitioners in models [77]. Usability of solution is another major challenge [44] underlining the importance of participatory design. Hospitals need to create an enabling infrastructure [86] to improve acceptance. It requires to be transparent on the limitations of these technologies [51, 103], to train and educate clinicians on BDA [86, 95], involve them in the design of solutions [55] and ensure they understand the gains [110]. Change teams should be considered to engage in peer learning [86] and educate staffs on changes induced by BDA [48].

##### Challenges for hospital to invest in BDA strategies

From a management perspective, investing in BDA is an opportunity to become a data-driven organization. Data-driven hospitals are built on the institutionalized network of technology, an analytics team and the administrative and clinical decision makers [109]. Large groups of hospitals, such as the Veterans health administration, Kaiser Permanente [108] and UCLA [96] have engaged in this complex process with some success.

The main challenge for hospitals investing in BDA strategies is to balance costs and benefits of BDA implementation and use [35].

A set of economic challenges (*n*=13) question the viability of BDA investments. The development of a BDA platform [68], the integration of data sources [37], their maintenance [95] and their analysis [87] are all heavy direct costs. Indirect costs can also be very significant as professionals need to invest time in solution development, which impacts clinical resources usage, and consequently, activity level and revenues [68]. If the costs are well defined, the benefits of investing in BDA are unclear and need to be assessed [89]. Our research sample underlines the difficulty to measure the impacts generated by the use of BDA. As such, calculating the ROI is only possible a posteriori, letting hospitals confronted with uncertainty. If hospitals are expected to invest in BDA, few incentive programs reward the use of BDA solutions financially [30]. There is a strong need for advocacy and lobbying to build political partnerships to adopt new approaches and support the emergence of incentives [79].

Beyond these economic considerations, management challenges (*n*=11) may prevent hospitals from succeeding in BDA projects. Investing in technological platforms is not enough [48]. Developing BDA strategies require to clearly define how and where to apply BDA, and to determin what the value targets  are,  which in turn, requires a strong engagement of the leadership team to develop a data-driven culture [86] and to align all contributors to the value chain [48]. Lack of such a culture could be detrimental to identifying and generating potential values of BDA.

## Discussion

In our scoping review, we aimed to explore how value is created from the use of BDA in hospitals. We found that if the use of BDA in hospitals has gained interest in the research community, most of the work done on the subject is technology-focused and has as main goals to demonstrate the relevance of BDA to solve medical or organizational challenges in hospitals, or the feasibility and validity of BDA solutions. Apart from a handful of references, value creation is approached as a by-product of BDA-driven knowledge generation and not a primary research objective.

From our review, there is evidence on the versatility of BDA to create value for hospitals. BDA can contribute to reach a large scope of value targets in all of hospital activity domains, and it can do so in a myriad of ways. We observe that value creation from BDA relies on a combinatory approach of resources, capabilities and mechanisms. BDAC are developed from the combination of data assets, technologies, techniques and skills, while knowledge is generated from the combination of BDACs. Hospital managers are facing countless possibilities to approach knowledge generation and address value-creating needs. Their challenge is to properly orchestrate resources to develop a set of BDACs that can support the right value creation mechanisms, with the objective of turning these capabilities into core competencies [111] which will eventually drive value creation and competitive advantage.

These outlooks on the value creation potential of BDA are particularly interesting as hospitals are exposed to internal and external pressures from regulatory changes, innovation or professional dynamics to which they need to adapt [112]. However, expectations regarding the realization of benefits from the use of BDA [6, 113] are far from being reflected in the literature. We observe a significant gap between the value creation potential of BDA solutions in hospital and their actual impacts. Evidence on the difficulties to realize value from investments made in BDAC building can be found applying the RBV’s Value-Rarity-Imitability-Organization (VRIO) framework [114]. There is no doubt that BDAC are valuable, rare and difficult to imitate. They are valuable as they enable hospitals to generate knowledge that can meet value creating needs. They are rare as data resources are, in most cases, hospital-specific [34], as skills are difficult to aggregate [30, 38, 92] and results generated from BDA solutions are often non-generalizable [44, 56, 115] from one context to the other. Finally, they are difficult to imitate as they are path-dependent, relying on previous HIT investments [51] and data collection practices, and socially complex as they cannot be managed in a systematic way [48] with each hospital leveraging its BDAC to adapt a specific context. However, in most articles reviewed, BDAC lack organizational embeddedness which is the fourth dimension of the VRIO framework. This lack of organizational embeddedness is observed at different levels. At the micro level, BDAC are dependent on individual practices. The generation of valuable knowledge can be hindered by poor data collection practices [66, 101] and inconsistent use of medical terminologies [49]. At the meso level, the lack of budget dedicated to BDA initiatives [68, 81], of change management to promote the cross-functional efforts needed [30, 86], of data culture in the organization [48, 95], can negatively impact the ability to develop BDAC, the value of the knowledge generated from BDAC or the acceptance and assimilation of this knowledge [36]. Finally, at the macro level, the lack of incentives to share data or invest in BDAC [42, 99] and the lack of institutional recognition of BDA-generated knowledge can factor in the realization of expected benefits. These organizational factors are as many weak links that can derail the value creation process from BDAC despite significant investments. It underlines that if BDAC can be valuable sources of knowledge, they cannot create value in isolation. They need to be combined harmoniously with other resources and capabilities to realize their potential and deliver on the value proposition hospitals are investing in [114, 116, 117].

While most of current research on BDA in hospitals is tactical, focusing on technological and technical dimensions and on narrow applications of BDA solutions, it appears essential to draw the attention of researchers and practitioners on strategic challenges. The debate should not stay on the potential of BDA applications and their ability to generate knowledge, but on how hospitals should get organized to acquire, process and use knowledge generated from BDAC while minimizing the weak links in the value creation process. The strategic challenges faced by hospitals are twofold: 1) they need to align their BDA strategy with hospitals' long term views, 2) they need to adapt their organizational capabilities to be able to move along the value creation process.

From an alignment perspective, if the development and implementation of BDAC is IT-oriented, given the costs and complexity of the operation, hospitals BDA strategy must be aligned with the institutions’ strategy and must be integrated in it. Managers need to combine mechanisms to drive this alignment. These mechanisms can be, among others, governance, procedures, data cultures.

From an organizational perspective, hospitals need to develop the capabilities that will enable them to explore knowledge generation opportunities, recognize relevant knowledge and assimilate it into their processes to generate impacts. The organization has to be able to articulate knowledge generation and assimilation.

The challenge for hospital managers is to define a context in which knowledge generation meet the objectives of the organization. This perspective is not covered in the existing literature. Empirical studies on how hospitals align their BDA and organizational strategies, and develop their organizational capabilities to create value are needed. Given the characteristics of BDA, the goal of hospitals professionals should be to generate impact from BDAC while minimizing the costs of development and appropriation.

## Strengths and limitations

Despite the growing interest of hospital managers for BDA and the significant investments made, there has been no review using a systematic search strategy focused on BDA applications in hospitals. Our systematic approach has enabled us to narrow down the analysis of value creation from BDA to the single provider perspective, hence differentiating our work from the existing sector-wide research. We also consider the use of an analytic framework as a major asset for conducting this review, especially in the process of data extraction. It allowed us to examine the path-to-value and its different components in a detailed yet systematic way. It was found essential to review BDA applications from very diverse settings, diversity which we decided to preserve by not restricting our search strategy to country of authorship.

Despite our efforts to ensure objectivity, accuracy and validity of our research, the latest still presents limitations. From a theoretical perspective, if the RBV is a largely accepted theory in strategic management, its use in healthcare is limited empirically although appearing promising [118]. The main reproach made by scholars is that publicly-funded organizations are more complex than private ones. However, we believe that even most hospitals are non-for-profit, they fundamentally compete to gain access to resources (patients, activity, healthcare professionals/skills, funding/investments) that determine their position in the system. If imperfect, high-level RBV concept can help understand the determinants of success for hospitals’ investments in BDA.

From a practical perspective, we limited our search to two databases: SCOPUS and Web of Sciences. Even though they are the largest multidisciplinary databases, the two most searched for research [29] and embed other databases such as Medline, they only cover part of the research produced.

Our keywords may have excluded articles that could have been of relevance to our review. Finally, as assessing quality is not a primary objective of a theoretical review, we did not focus on the robustness of selected references

## Conclusion

This scoping review is the first study that explores how value is created from BDA in hospitals. Its contribution is twofold. On the one hand, it confirms the versatility and value creation potential of BDA capabilities in hospital. Articles reviewed demonstrate the technological feasibility of BDA-driven knowledge generation solutions that can address value creation needs in all of hospitals’ main activity domains. On the other hand, it points at a glaring gap between the value creation potential of BDA solutions and their actual impacts. Availability of BDA capabilities and BDA-generated knowledge are necessary and yet insufficient conditions for value creation. In many cases, BDA capabilities are built independently of organizational characteristics and goals and are unable to trigger the value creation mechanisms that will enable hospitals move along the path-to-value.

The configuring of strategies, technologies and organizational capabilities around which the movement towards value realization is orchestrated should become a priority area for research. In that sense, we encourage future empirical work on the mechanisms that can drive the alignment of BDA and organizational strategies, and on the development of organizational capabilities required to support knowledge generation and assimilation in ways that support the realization of BDA potential.

We hope this review will encourage hospital professionals to reflect upon the factors needed to develop BDA strategies and that our analytical framework will be the basis of a practical tool to explore facilitators and barriers in the development of BDA in hospitals.

## Supplementary Information

**Additional file 1.**

## Data Availability

The datasets analyzed in this study are available from the corresponding author on request.
