# Supplementary material for: The path from big data analytics capabilities to value in hospitals: a scoping review
Source: BMC Health Serv Res. 2022 Jan 31;22:134. doi: 10.1186/s12913-021-07332-0 (PMC8805378; doi:10.1186/s12913-021-07332-0)
Supplement: Supplementary file 1 — Additional file 1. [file 12913_2021_7332_MOESM1_ESM.docx]

**The path from big data analytics capabilities to value in hospitals: a scoping review**

#

# **Appendix**

**Supplementary Table 1.** PRISMA scoping review checklist

| **SECTION** | **ITEM** | **PRISMA-ScR CHECKLIST ITEM** | **REPORTED ON PAGE #** |
| --- | --- | --- | --- |
| **TITLE** | | | |
| Title | 1 | Identify the report as a scoping review. | p. 1 |
| **ABSTRACT** | | | |
| Structured summary | 2 | Provide a structured summary that includes (as applicable): background, objectives, eligibility criteria, sources of evidence, charting methods, results, and conclusions that relate to the review questions and objectives. | p. 1-2 |
| **INTRODUCTION’** | | | |
| Rationale | 3 | Describe the rationale for the review in the context of what is already known. Explain why the review questions/objectives lend themselves to a scoping review approach. | p. 4-5 |
| Objectives | 4 | Provide an explicit statement of the questions and objectives being addressed with reference to their key elements (e.g., population or participants, concepts, and context) or other relevant key elements used to conceptualize the review questions and/or objectives. | p. 4-5, 7 |
| **METHODS** | | | |
| Protocol and registration | 5 | Indicate whether a review protocol exists; state if and where it can be accessed (e.g., a Web address); and if available, provide registration information, including the registration number. | / |
| Eligibility criteria | 6 | Specify characteristics of the sources of evidence used as eligibility criteria (e.g., years considered, language, and publication status), and provide a rationale. | p. 8-9 |
| Information sources* | 7 | Describe all information sources in the search (e.g., databases with dates of coverage and contact with authors to identify additional sources), as well as the date the most recent search was executed. | p. 8 |
| Search | 8 | Present the full electronic search strategy for at least 1 database, including any limits used, such that it could be repeated. | p. 8 |
| Selection of sources of evidence† | 9 | State the process for selecting sources of evidence (i.e., screening and eligibility) included in the scoping review. | p. 8-9 |
| Data charting process‡ | 10 | Describe the methods of charting data from the included sources of evidence (e.g., calibrated forms or forms that have been tested by the team before their use, and whether data charting was done independently or in duplicate) and any processes for obtaining and confirming data from investigators. | p. 9 |
| Data items | 11 | List and define all variables for which data were sought and any assumptions and simplifications made. | p. 5-6, 9 |
| Critical appraisal of individual sources of evidence§ | 12 | If done, provide a rationale for conducting a critical appraisal of included sources of evidence; describe the methods used and how this information was used in any data synthesis (if appropriate). | / |
| Synthesis of results | 13 | Describe the methods of handling and summarizing the data that were charted. | p. 9 |
| **RESULTS** | | | |
| Selection of sources of evidence | 14 | Give numbers of sources of evidence screened, assessed for eligibility, and included in the review, with reasons for exclusions at each stage, ideally using a flow diagram. | p. 10 |
| Characteristics of sources of evidence | 15 | For each source of evidence, present characteristics for which data were charted and provide the citations. | p. 10-27 |
| Critical appraisal within sources of evidence | 16 | If done, present data on critical appraisal of included sources of evidence (see item 12). | / |
| Results of individual sources of evidence | 17 | For each included source of evidence, present the relevant data that were charted that relate to the review questions and objectives. | Appendix (ST2, 3; 4) |
| Synthesis of results | 18 | Summarize and/or present the charting results as they relate to the review questions and objectives. | p. 10-27 |
| **DISCUSSION** | | | |
| Summary of evidence | 19 | Summarize the main results (including an overview of concepts, themes, and types of evidence available), link to the review questions and objectives, and consider the relevance to key groups. | p. 28-29 |
| Limitations | 20 | Discuss the limitations of the scoping review process. | p. 31 |
| Conclusions | 21 | Provide a general interpretation of the results with respect to the review questions and objectives, as well as potential implications and/or next steps. | p. 32-33 |
| **FUNDING** | | | |
| Funding | 22 | Describe sources of funding for the included sources of evidence, as well as sources of funding for the scoping review. Describe the role of the funders of the scoping review. | p. 42 |

**Supplementary Table 2.** Description of the path-to-value from BDAC (n = 94)

| **References** | **BDA capabilities** | **Value creation mechanisms** | **Targets (FT, VT)** | **Benefits** | **Challenges** |
| --- | --- | --- | --- | --- | --- |
| (Al’Aref et al. 2019) (1) | **2. Analysis**  Predictive | **VCM2**. Discovery and experimentation  **VCM3**. Segmentation | **VT1** - Decision making:  C - Risk detection | **B1. Operational:**  Quality improvement:  *Clinical decision* | **C1. Generation of valuable knowledge**:  Data quality  Methodological challenges |
| (Alnsour, Hadidi, et Singh 2019) (2) | **2. Analysis:**  Predictive | **VCM2**. Discovery and experimentation  **VCM3**. Segmentation | **VT1** - Decision making:  C - Risk detection  **VT2** - Innovation:  C - Preventative medicine | **B1. Operational:**  Quality improvement:  *Clinical decision*  *Clinical outcomes*  **B2. Organizational:**  Support learning and skills development  **B3. Managerial**  Resource management | **C1. Generation of valuable knowledge:**  Access to data |
| (An et al. 2018) (3) | **2. Analysis:**  Analytical  Predictive | **VCM1**. Process and outcomes transparency  **VCM2**. Discovery and experimentation  **VCM3**. Segmentation | **VT1** - Decision making:  C - Risk detection  **VT2** - Innovation:  C - Precision medicine | **B1. Operational:**  Cost reduction:  *Unnecessary care*  Quality improvement:  *Clinical decision*  *Clinical outcomes*  **B2. Organizational:**  Change work patterns | **C1. Generation of valuable knowledge**:  Access to data  Data quality  Techno related challenges  **C2. Transforming knowledge into actions**:  Acceptance |
| (Anderson et Chang 2015) (4) | **1. Acquisition:** Traceability  **2. Analysis:**  Analytical | **VCM1**. Process and outcomes transparency  **VCM2**. Discovery and experimentation | **VT1** - Decision making:  M - Assess activities | **B1. Operational:**  Productivity gains  Service improvement:  *For patients*  **B3. Managerial**  Performance improvement | **C1. Generation of valuable knowledge:**  Access to data  **C2. Transforming knowledge into actions**:  Acceptance |
| (Avati et al. 2018) (5) | **2. Analysis:**  Predictive  **3. Interpretation:** Decision support | **VCM2**. Discovery and experimentation  **VCM3**. Segmentation  **VCM5**. Monitoring | **VT1** - Decision making:  C - Risk detection  M - Resource allocation  **VT2** - Innovation:  C - Precision medicine | **B1. Operational:**  Productivity gains  Quality improvement:  *Clinical decision*  **B2. Organizational:**  Change work patterns | **C1. Generation of valuable knowledge:**  Access to data  Techno related challenges  **C2. Transforming knowledge into actions**:  Acceptance |
| (Baechle et Agarwal 2017)(6) | **2. Analysis:**  Analytical  **3. Interpretation:** Decision support | **VCM1**. Process and outcomes transparency  **VCM2**. Discovery and experimentation  **VCM3**. Segmentation  **VCM5**. Monitoring | **VT1** - Decision making:  M - Assess activities  **VT2** - Innovation:  C - Preventative medicine  **VT3** - Performance:  C - Patient flow | **B1. Operational:**  Cost reduction:  *Other*  **B2. Organizational:**  Change work patterns  Communication and collaboration  **B3. Managerial**  Resource management |  |
| (Baghapour et al. 2018) (7) | **1. Acquisition:** Interoperability  **2. Analysis:**  Analytical  **3. Interpretation:** Decision support | **VCM1**. Process and outcomes transparency  **VCM2**. Discovery and experimentation | **VT3** - Performance:  M - Operations management | **B1. Operational:**  Cost reduction:  *Other*  **B3. Managerial**  Resource management  Decision-making and planning | **C1. Generation of valuable knowledge:**  Data quality  Techno related challenges |
| (Bouzillé et al. 2019) (8) | **1. Acquisition:** Traceability  **2. Analysis:**  Analytical  **3. Interpretation:** Decision support | **VCM1**. Process and outcomes transparency  **VCM2**. Discovery and experimentation  **VCM5**. Monitoring | **VT2** - Innovation:  C - Preventative medicine | **B1. Operational:**  Service improvement:  *For HCPs*  **B2. Organizational:**  Support learning and skills development | **C1. Generation of valuable knowledge:**  Access to data  Techno related challenges  **C2. Transforming knowledge into actions:**  Acceptance |
| (Bygstad et al. 2020) (9) | **1. Acquisition:** Traceability  Interoperability  **2. Analysis:**  Analytical  **3. Interpretation:** Decision support | **VCM1**. Process and outcomes transparency  **VCM2**. Discovery and experimentation | **VT1** - Decision making:  M - Assess activities  **VT3** - Performance:  C - Patient flow | **B2. Organizational:**  Change work patterns  **B3. Managerial**  Decision-making and planning  Performance improvement | **C1. Generation of valuable knowledge:**  Access to data  Techno related challenges  **C2. Transforming knowledge into actions:**  Limited awareness  Complexity  Acceptance |
| (Calcaterra et al. 2018) (10) | **1. Acquisition:** Traceability  **2. Analysis:**  Predictive | **VCM2**. Discovery and experimentation  **VCM3**. Segmentation  **VCM4**. Predict | **VT2** - Innovation:  C - Preventative medicine | **B1. Operational:**  Quality improvement:  *Clinical decision*  **B2. Organizational:**  Support learning and skills development | **C1. Generation of valuable knowledge:**  Access to data  Data quality  Techno related challenges  **C2. Transforming knowledge into actions:**  Limited awareness  **C3. Challenges for hospitals to invest in BDA strategies:**  Economic challenges |
| (J. Chen et al. 2016) (11) | **1. Acquisition:** Traceability  **2. Analysis:**  Predictive  **3. Interpretation:** Decision support | **VCM1**. Process and outcomes transparency  **VCM2**. Discovery and experimentation | **VT3** - Performance:  C - Patient flow | **B1. Operational:**  Productivity gains  **B2. Organizational:**  Change work patterns | **C1. Generation of valuable knowledge:**  Techno related challenges |
| (Z. Chen et al. 2018) (12) | **2. Analysis:**  Analytical  Predictive | **VCM2**. Discovery and experimentation  **VCM3**. Segmentation  **VCM5**. Monitoring | **VT1** - Decision making:  C - Diagnostic | **B1. Operational:**  Cost reduction:  *Unnecessary care*  Quality improvement:  *Clinical decision*  Service improvement:  *For HCPs* | **C1. Generation of valuable knowledge:**  Access to data  Techno related challenges |
| (Cheng et Kuo 2016) (13) | **1. Acquisition:** Traceability  Interoperability  **2. Analysis:**  Analytical  **3. Interpretation:** Decision support | **VCM1**. Process and outcomes transparency  **VCM2**. Discovery and experimentation  **VCM5**. Monitoring | **VT1** - Decision making:  M - Resource allocation  **VT3** - Performance:  C - Patient flow  M - Operations management | **B1. Operational:**  Cost reduction:  *Other*  Quality improvement:  *Clinical outcomes*  **B2. Organizational:**  Support learning and skills development  Change work patterns  **B3. Managerial**  Resource management  Decision-making and planning | **C1. Generation of valuable knowledge:**  Techno related challenges  **C2. Transforming knowledge into actions:**  Acceptance |
| (Clarke et al. 2015) (14) | **1. Acquisition:** Traceability  **2. Analysis:**  Analytical  **3. Interpretation:** Decision support | **VCM1**. Process and outcomes transparency  **VCM2**. Discovery and experimentation | **VT1** - Decision making:  M - Assess activities  **VT2** - Innovation:  C - Precision medicine  C - Preventative medicine  M - Adapt strategies  **VT3** - Performance:  C - Patient flow | **B1. Operational:**  Quality improvement:  *Clinical decision*  *Clinical outcomes*  Service improvement:  *For HCPs*  **B2. Organizational:**  Support learning and skills development  **B4. Strategic:**  Differentiation  Growth  Positioning  Resources | **C2. Transforming knowledge into actions:**  Complexity  Acceptance  **C3. Challenges for hospitals to invest in BDA strategies:**  Management challenges |
| (Clifford et al. 2018) (15) | **1. Acquisition:** Traceability  **2. Analysis:**  Analytical  **3. Interpretation:** Decision support | **VCM1**. Process and outcomes transparency  **VCM2**. Discovery and experimentation  **VCM5**. Monitoring | **VT1** - Decision making:  M - Assess activities  **VT2** - Innovation:  C - Preventative medicine  M - Adapt strategies | **B1. Operational:**  Productivity gains  **B3. Managerial**  Performance improvement  **B4. Strategic:**  Differentiation | **C1. Generation of valuable knowledge:**  Access to data  Techno related challenges  Lack of talents |
| (Cobb et al. 2018) (16) | **1. Acquisition:** Traceability  **2. Analysis:**  Analytical | **VCM1**. Process and outcomes transparency  **VCM2**. Discovery and experimentation | **VT1** - Decision making:  M - Assess activities | **B3. Managerial**  Resource management  Decision-making and planning | **C1. Generation of valuable knowledge:**  Access to data  Data quality |
| (Cresswell et al. 2016) (17) | **2. Analysis:**  Analytical  Predictive  **3. Interpretation:** Decision support | **VCM2**. Discovery and experimentation  **VCM3**. Segmentation  **VCM4**. Predict | **VT1 -** Decision making:  M - Assess activities  **VT2** – Innovation  M - Adapt strategies | **B1. Operational:**  Productivity gains  Cost reduction:  *Unnecessary care*  Quality improvement:  *Clinical decision*  **B2. Organizational:**  Support learning and skills development  **B3. Managerial**  Resource management  Performance improvement  **B4. Strategic:**  Differentiation | **C1. Generation of valuable knowledge:**  Lack of talents  **C2. Transforming knowledge into actions:**  Acceptance  **C3. Challenges for hospitals to invest in BDA strategies:**  Economic challenges  Management challenges |
| (Dagliati et al. 2018) (18) | **1. Acquisition:**  Traceability  Interoperability  **2. Analysis:**  Analytical  Predictive  **3. Interpretation:** Decision support | **VCM1**. Process and outcomes transparency  **VCM2**. Discovery and experimentation  **VCM4**. Predict | **VT2** - Innovation:  C - Preventative medicine | **B1. Operational:**  Productivity gains  Cost reduction:  *Other*  Quality improvement:  *Clinical decision*  **B2. Organizational:**  Support learning and skills development  **B3. Managerial**  Performance improvement  **B4. Strategic:**  Differentiation | **C1. Generation of valuable knowledge:**  Access to data  Data quality  Methodological challenges  **C3. Challenges for hospitals to invest in BDA strategies:**  Management challenges |
| (Damle et Alavi 2016) (19) | **1. Acquisition:** Traceability  Interoperability  **2. Analysis:**  Analytical | **VCM1**. Process and outcomes transparency  **VCM2**. Discovery and experimentation | **VT3** - Performance:  R - Research performance | **B3. Managerial**  Resource management  Performance improvement  **B4. Strategic:**  Innovation | **C1. Generation of valuable knowledge:**  Access to data  Techno related challenges  Methodological challenges  **C2. Transforming knowledge into actions:**  Acceptance |
| (Danner et al. 2017) (20) | **2. Analysis:**  Predictive | **VCM2**. Discovery and experimentation  **VCM3**. Segmentation | **VT1** - Decision making:  C – Diagnostic  **VT2** - Innovation:  C - Precision medicine | **B1. Operational:**  Quality improvement:  *Clinical decision*  *Clinical outcomes* |  |
| (Delahanty, Kaufman, et Jones 2018) (21) | 1. Acquisition: Interoperability  2. Analysis: Predictive  3. Interpretation: Decision support | **VCM2**. Discovery and experimentation  **VCM3**. Segmentation  **VCM4**. Predict | **VT1** - Decision making:  C - Risk detection  **VT2** - Innovation:  C - Precision medicine | **B1. Operational:**  Productivity gains  Cost reduction:  *Other*  Quality improvement:  *Clinical* outcomes  Service improvement:  For HCPs  **B4. Strategic:** | **C1. Generation of valuable knowledge:**  Access to data  Data quality  Lack of talents  **C2. Transforming knowledge into actions:**  Complexity  Acceptance  **C3. Challenges for hospitals to invest in BDA strategies:**  Economic challenges |
| (Desai, Roberts, et Wilkerson 2016) (22) | **1. Acquisition:** Traceability  **2. Analysis:**  Analytical  **3. Interpretation:** Decision support | **VCM1**. Process and outcomes transparency  **VCM2**. Discovery and experimentation | **VT2** - Innovation:  M - Adapt strategies | **B3. Managerial**  Resource management  Decision-making and planning  **B4. Strategic:**  Innovation | **C1. Generation of valuable knowledge:**  Access to data  Lack of talents |
| (Dreyfus et al. 2018) (23) | **1. Acquisition:** Traceability  **2. Analysis:**  Analytical | **VCM1**. Process and outcomes transparency  **VCM2**. Discovery and experimentation  **VCM3**. Segmentation | **VT2** - Innovation:  C - Preventative medicine | **B1. Operational:**  Quality improvement:  *Clinical* *decision*  **B2. Organizational:**  Change work patterns  **B3. Managerial**  Performance improvement  **B4. Strategic:**  Positioning  Influence | **C1. Generation of valuable knowledge:**  Access to data  Data quality  Techno related challenges  **C3. Challenges for hospitals to invest in BDA strategies:**  Economic challenges |
| (DuBay et al. 2019) (24) | **1. Acquisition:** Interoperability  **2. Analysis:**  Predictive | **VCM1**. Process and outcomes transparency  **VCM2**. Discovery and experimentation  **VCM3**. Segmentation  **VCM4**. Predict | **VT1** - Decision making:  C - Risk detection  **VT2** - Innovation:  C - Precision medicine | **B1. Operational:**  Cost reduction:  *Unnecessary care*  Quality improvement:  *Clinical decision*  **B3. Managerial**  Resource management | **C1. Generation of valuable knowledge:**  Access to data |
| (Elvira et al. 2018) (25) | **1. Acquisition:** Traceability  **2. Analysis:**  Predictive | **VCM1**. Process and outcomes transparency  **VCM2**. Discovery and experimentation  **VCM3**. Segmentation | **VT3** - Performance:  C - Patient flow | **B1. Operational:**  Productivity gains  Cost reduction:  *Other*  Service improvement:  *For patients*  **B3. Managerial**  Performance improvement | **C1. Generation of valuable knowledge:**  Access to data  Techno related challenges |
| (Ficheur et al. 2015) (26) | **2. Analysis:**  Analytical  **3. Interpretation:** Decision support | **VCM1**. Process and outcomes transparency  **VCM2**. Discovery and experimentation | **VT2** - Innovation:  C - Preventative medicine | **B1. Operational:**  Productivity gains | **C1. Generation of valuable knowledge:**  Access to data  Data quality  **C2. Transforming knowledge into actions:**  Acceptance |
| (Foster et al. 2018) (27) | **1. Acquisition:** Interoperability  **2. Analysis:**  Analytical  **3. Interpretation:** Decision support | **VCM1**. Process and outcomes transparency  **VCM2**. Discovery and experimentation | **VT1** - Decision making:  M - Assess activities  M - Resource allocation | **B3. Managerial**  Resource management  Decision-making and planning  Performance improvement | **C1. Generation of valuable knowledge:**  Access to data  Techno related challenges  **C2. Transforming knowledge into actions:**  Complexity  Acceptance |
| (Genevès et al. 2018) (28) | **1. Acquisition:** Traceability  **2. Analysis:**  Predictive | **VCM2**. Discovery and experimentation  **VCM3**. Segmentation  **VCM5**. Monitoring | **VT1** - Decision making:  C - Risk detection  **VT2** - Innovation:  C - Preventative medicine | **B1. Operational:**  Cost reduction:  Unnecessary care  *Other*  **B2. Organizational:**  Change work patterns  **B3. Managerial**  Resource management | **C1. Generation of valuable knowledge:**  Techno related challenges |
| (Godinho et al. 2019) (29) | **1. Acquisition:** Interoperability  **2. Analysis:**  Analytical  **3. Interpretation:** Decision support | **VCM1**. Process and outcomes transparency  **VCM2**. Discovery and experimentation | **VT3** - Performance:  R - Research performance | **B1. Operational:**  Productivity gains  Quality improvement: *Clinical outcomes*  Service improvement:  *For HCPs*  **B2. Organizational:**  Support learning and skills development | **C1. Generation of valuable knowledge:**  Data quality  Techno related challenges  **C3. Challenges for hospitals to invest in BDA strategies:**  Economic challenges |
| (Golas et al. 2018) (30) | **1. Acquisition:**  Traceability  Interoperability  **2. Analysis:**  Analytical  Predictive | **VCM1**. Process and outcomes transparency  **VCM2**. Discovery and experimentation  **VCM3**. Segmentation | **VT1** - Decision making:  C - Risk detection  **VT2** - Innovation:  C - Precision medicine | **B1. Operational:**  Cost reduction:  *Reduce readmissions*  Quality improvement:  *Clinical decision*  *Clinical outcomes*  **B2. Organizational:**  Change work patterns | **C1. Generation of valuable knowledge:**  Access to data  Techno related challenges |
| (Gonçalves et al. 2018) (31) | **1. Acquisition:** Traceability  **2. Analysis:**  Analytical | **VCM2**. Discovery and experimentation | **VT3** - Performance:  C - Patient flow | **B2. Organizational:**  Change work patterns |  |
| (Guan et al. 2017) (32) | **1. Acquisition:** Traceability  **2. Analysis:**  Analytical  Predictive  **3. Interpretation:** Decision support | **VCM1**. Process and outcomes transparency  **VCM2**. Discovery and experimentation  **VCM4**. Predict | **VT1** - Decision making:  M - Resource allocation  **VT3** - Performance:  M - Operations management | **B1. Operational:**  Cost reduction:  *Other*  Quality improvement:  *Clinical outcomes*  **B3. Managerial**  Resource management | **C1. Generation of valuable knowledge:**  Access to data  Data quality |
| (Halamka 2014) (33) | **2. Analysis:**  Predictive | **VCM2**. Discovery and experimentation | **VT1** - Decision making:  M - Assess activities | **B1. Operational:**  Productivity gains  Cost reduction:  *Other*  **B2. Organizational:**  Support learning and skills development  Change work patterns  Communication and collaboration  **B3. Managerial**  Resource management  Decision-making and planning  **B4. Strategic:**  Differentiation | **C1. Generation of valuable knowledge:**  Data quality  Lack of talents |
| (Hendricks 2019) (34) | **2. Analysis:**  Analytical | **VCM2**. Discovery and experimentation  **VCM5**. Monitoring | **VT1** - Decision making:  M - Assess activities  R - Hypothesis setting  **VT2** - Innovation:  R - New research tools  **VT3** - Performance:  C - Patient flow | **B2. Organizational:**  Support learning and skills development  **B3. Managerial**  Decision-making and planning | **C1. Generation of valuable knowledge:**  Access to data  Techno related challenges  **C2. Transforming knowledge into actions:**  Acceptance |
| (Hewner, Sullivan, et Yu 2018) (35) | **1. Acquisition:**  Traceability  Interoperability  **2. Analysis:**  Analytical  **3. Interpretation:** Decision support | **VCM2**. Discovery and experimentation  **VCM3**. Segmentation  **VCM4**. Predict | **VT2** - Innovation:  C - Preventative medicine  **VT3** - Performance:  C - Patient flow | **B1. Operational:**  Revenue growth  Productivity gains  Cost reduction:  *Reduce readmissions*  Service improvement:  *For patients*  *For HCPs*  **B2. Organizational:**  Change work patterns  **B4. Strategic:**  Growth | **C1. Generation of valuable knowledge:**  Techno related challenges  **C2. Transforming knowledge into actions:**  Complexity  **C3. Challenges for hospitals to invest in BDA strategies:**  Management challenges |
| (Ho et al. 2019) (36) | **2. Analysis:**  Analytical  Predictive  **3. Interpretation:** Decision support | **VCM2**. Discovery and experimentation  **VCM4**. Predict  **VCM5**. Monitoring | **VT1** - Decision making:  M - Resource allocation  **VT3** - Performance:  C - Patient flow | **B1. Operational:**  Quality improvement:  *Clinical decision*  Service improvement:  *For patients*  **B2. Organizational**:  Change work patterns  **B3. Managerial**  Resource management | **C1. Generation of valuable knowledge:**  Data quality  Techno related challenges |
| (Y. Hu et al. 2018) (37) | **2. Analysis:**  Predictive  **3. Interpretation:** Decision support | **VCM2**. Discovery and experimentation  **VCM3**. Segmentation  **VCM4**. Predict | **VT1** - Decision making:  C – Diagnostic  **VT3** - Performance:  C - Patient flow | **B1. Operational:**  Productivity gains  Quality improvement:  *Clinical decision*  Service improvement:  *For HCPs* | **C1. Generation of valuable knowledge:**  Data quality |
| (S.-Y. Hu et al. 2019) (38)h | **2. Analysis:**  Analytical  Predictive | **VCM2**. Discovery and experimentation  **VCM3**. Segmentation  **VCM4**. Predict | **VT1** - Decision making:  C – Diagnostic  **VT2** - Innovation:  C - Precision medicine | **B1. Operational:**  Cost reduction:  *Unnecessary care*  *Other*  Quality improvement:  *Clinical decision*  *Clinical outcomes*  **B2. Organizational:**  Support learning and skills development | **C1. Generation of valuable knowledge:**  Access to data  Techno related challenges  **C2. Transforming knowledge into actions:**  Complexity  Acceptance |
| (Ibanez-Sanchez et al. 2019) (39) | **1. Acquisition:** Traceability  **2. Analysis:**  Analytical | **VCM1**. Process and outcomes transparency  **VCM2**. Discovery and experimentation | **VT2** - Innovation:  M - Adapt strategies  **VT3** - Performance:  C - Patient flow | **B1. Operational:**  Productivity gains  Quality improvement:  *Clinical outcomes*  Service improvement:  *For HCPs*  **B2. Organizational:**  Change work patterns  Communication and collaboration  **B3. Managerial**  Resource management  **B4. Strategic:**  Growth | **C1. Generation of valuable knowledge:**  Techno related challenges  **C2. Transforming knowledge into actions:**  Acceptance |
| (Janke et al. 2016) (40) | **2. Analysis:**  Analytical  Predictive  **3. Interpretation:** Decision support | **VCM1**. Process and outcomes transparency  **VCM2**. Discovery and experimentation  **VCM5**. Monitoring | **VT2** - Innovation:  C - Precision medicine  C - Preventative medicine  **VT3** - Performance:  C - Patient flow | **B1. Operational:**  Cost reduction:  *Other*  Quality improvement:  *Clinical decision*  Service improvement:  *For HCPs*  **B4. Strategic:** | **C1. Generation of valuable knowledge:**  Data quality  Methodological challenges  Lack of talents  **C2. Transforming knowledge into actions:**  Acceptance  **C3. Challenges for hospitals to invest in BDA strategies:**  Economic challenges |
| (Johnson, Hall, et Hulme 2016) (41) | **2. Analysis:**  Analytical | **VCM1**. Process and outcomes transparency  **VCM2**. Discovery and experimentation | **VT1** - Decision making:  M - Resource allocation  R - Hypothesis setting  **VT2** - Innovation:  R - New research tools | **B1. Operational:**  Productivity gains  **B2. Organizational:**  Change work patterns  Communication and collaboration  **B3. Managerial**  Resource management  **B4. Strategic:**  Growth | **C1. Generation of valuable knowledge:**  Lack of talents  **C2. Transforming knowledge into actions:**  Complexity |
| (Kang, Seo, et Kim 2019) (42) | **2. Analysis:**  Analytical | **VCM2**. Discovery and experimentation  **VCM3**. Segmentation | **VT1** - Decision making:  C - Risk detection  **VT2** - Innovation:  C - Preventative medicine | **B1. Operational:**  Cost reduction:  *Other*  **B2. Organizational:**  Change work patterns | **C1. Generation of valuable knowledge:**  Data quality  **C2. Transforming knowledge into actions:**  Complexity  **C3. Challenges for hospitals to invest in BDA strategies:**  Economic challenges |
| (Karanastasis et al. 2019) (43) | **1. Acquisition:**  Traceability  Interoperability  **2. Analysis:**  Analytical  **3. Interpretation:** Decision support | **VCM2**. Discovery and experimentation | **VT3** - Performance:  R - Research performance | **B1. Operational:**  Productivity gains  Quality improvement:  *Clinical decision*  **B2. Organizational:**  Communication and collaboration  **B3. Managerial**  Performance improvement  **B4. Strategic:**  Differentiation  Growth | **C1. Generation of valuable knowledge:**  Access to data  Techno related challenges  **C2. Transforming knowledge into actions:**  Complexity  **C3. Challenges for hospitals to invest in BDA strategies:**  Economic challenges  Management challenges |
| (Karnuta et al. 2019) (44) | **1. Acquisition:**  Traceability  **2. Analysis:**  Predictive | **VCM1**. Process and outcomes transparency  **VCM2**. Discovery and experimentation  **VCM3**. Segmentation | **VT1** - Decision making:  M - Assess activities  **VT2** - Innovation:  C - Precision medicine | **B2. Organizational:**  Change work patterns  **B3. Managerial**  Resource management  **B4. Strategic:**  Differentiation  Positioning  Influence | **C1. Generation of valuable knowledge:**  Access to data  Lack of talents  **C3. Challenges for hospitals to invest in BDA strategies:**  Management challenges |
| (Kern, Reagin, et Reese 2016) (45) | **1. Acquisition:**  Traceability  Interoperability  **2. Analysis:**  Analytical  Predictive  **3. Interpretation:**  Decision support | **VCM1**. Process and outcomes transparency  **VCM2**. Discovery and experimentation  **VCM3**. Segmentation  **VCM5**. Monitoring | **VT2** - Innovation:  M - Adapt strategies | **B1. Operational:**  Cost reduction:  *Other*  Quality improvement:  *Clinical outcomes*  **B2. Organizational:**  Support learning and skills development  Change work patterns  Communication and collaboration  **B3. Managerial**  Resource management  Performance improvement  **B4. Strategic:**  Innovation  Positioning  Resources | **C1. Generation of valuable knowledge:**  Access to data  Data quality  Techno related challenges  Lack of talents  **C3. Challenges for hospitals to invest in BDA strategies:**  Economic challenges  Management challenges |
| (Khalifa 2015) (46) | **2. Analysis:**  Analytical | **VCM1**. Process and outcomes transparency  **VCM2**. Discovery and experimentation  **VCM3**. Segmentation | **VT1** - Decision making:  C - Risk detection  **VT3** - Performance:  C - Patient flow | **B1. Operational:**  Cost reduction:  *Unnecessary care*  Service improvement:  *For patients* |  |
| (Khalifa 2016) (47) | **1. Acquisition:**  Traceability  **2. Analysis:**  Analytical  **3. Interpretation:** Decision support | **VCM1**. Process and outcomes transparency  **VCM2**. Discovery and experimentation  **VCM3**. Segmentation  **VCM5**. Monitoring | **VT1** - Decision making:  M - Assess activities  **VT3** - Performance:  C - Patient flow | **B1. Operational:**  Productivity gains  Quality improvement:  *Clinical outcomes*  **B2. Organizational:**  Change work patterns  **B3. Managerial**  Resource management | **C1. Generation of valuable knowledge:**  Access to data  Techno related challenges |
| (Kim et al. 2019) (48) | **2. Analysis:**  Predictive  **3. Interpretation:** Decision support | **VCM2**. Discovery and experimentation  **VCM3**. Segmentation  **VCM5**. Monitoring | **VT2** - Innovation:  C - Precision medicine | **B1. Operational:**  Quality improvement:  *Clinical outcomes*  **B2. Organizational:**  Change work patterns  **B3. Managerial**  Resource management | **C1. Generation of valuable knowledge:**  Techno related challenges  Methodological challenges  **C2. Transforming knowledge into actions:**  Acceptance |
| (Krämer, Schreyögg, et Busse 2019) (49) | **2. Analysis:**  Analytical  Predictive  **3. Interpretation:**  Decision support | **VCM1**. Process and outcomes transparency  **VCM2**. Discovery and experimentation  **VCM3**. Segmentation | **VT2** - Innovation:  C - Precision medicine  **VT3** - Performance:  C - Patient flow | **B1. Operational:**  Productivity gains  **B2. Organizational:**  Change work patterns  **B3. Managerial**  Decision-making and planning  **B4. Strategic:**  Innovation | **C1. Generation of valuable knowledge:**  Data quality  Techno related challenges  Methodological challenges |
| (Kreuger et al. 2018) (50) | **1. Acquisition:** Traceability  Interoperability  **2. Analysis:**  Analytical | **VCM2**. Discovery and experimentation | **VT1** - Decision making:  C - Risk detection  **VT3** - Performance:  R - Research performance | **B1. Operational:**  Productivity gains | **C1. Generation of valuable knowledge:**  Access to data  Data quality  Techno related challenges  Methodological challenges |
| (Kuhajda 2016) (51) | **2. Analysis:**  Analytical  **3. Interpretation:** Decision support | **VCM2**. Discovery and experimentation  **VCM4**. Predict | **VT3** - Performance:  M - Operations management | **B1. Operational**:  Cost reduction:  *Other*  Service improvement:  *For HCPs*  **B3. Managerial**  Resource management | **C1. Generation of valuable knowledge:**  Access to data |
| (Leary et al. 2016) (52) | **1. Acquisition:**  Traceability  Interoperability  **2. Analysis:**  Analytical | **VCM2**. Discovery and experimentation | **VT1** - Decision making:  M - Resource allocation | **B1. Operational:**  Quality improvement:  Clinical outcomes  **B3. Managerial**  Resource management | **C1. Generation of valuable knowledge:**  Access to data  Data quality  Techno related challenges  **C3. Challenges for hospitals to invest in BDA strategies:**  Management challenges |
| (Ledieu 2018) (53) | **1. Acquisition:**  Traceability  **2. Analysis:**  Analytical  **3. Interpretation:**  Decision support | **VCM1**. Process and outcomes transparency  **VCM2**. Discovery and experimentation  **VCM5**. Monitoring | **VT2** - Innovation:  C - Preventative medicine | **B1. Operational:**  Quality improvement:  Clinical decision | **C1. Generation of valuable knowledge:**  Techno related challenges  Lack of talents  **C2. Transforming knowledge into actions:**  Acceptance |
| (Li et al. 2019) (54) | **1. Acquisition:**  Traceability  Interoperability  **2. Analysis:**  Analytical | **VCM2**. Discovery and experimentation | **VT1** - Decision making:  C – Diagnostic  **VT3** - Performance:  R - Research performance | **B3. Managerial**  Performance improvement | **C1. Generation of valuable knowledge:**  Access to data  Data quality  **C2. Transforming knowledge into actions:**  Acceptance |
| (Lodhi et al. 2017) (55) | **1. Acquisition:**  Traceability  Interoperability  **2. Analysis:**  Predictive | **VCM2**. Discovery and experimentation  **VCM3**. Segmentation | **VT2** - Innovation:  C - Preventative medicine  **VT3** - Performance:  C - Patient flow | **B1. Operational:**  Cost reduction:  *Reduce readmissions*  **B2. Organizational:**  Change work patterns  **B3. Managerial**  Resource management | **C1. Generation of valuable knowledge:**  Access to data  Data quality  **C2. Transforming knowledge into actions:**  Complexity |
| (Lodhi et al. 2015) (56) | **2. Analysis:**  Predictive  **3. Interpretation:**  Decision support | **VCM2**. Discovery and experimentation  **VCM3**. Segmentation | **VT2** - Innovation:  C - Precision medicine | **B1. Operational:**  Cost reduction:  *Unnecessary care*  Quality improvement:  *Clinical outcomes*  Service improvement:  *For HCPs*  **B2. Organizational:**  Change work patterns |  |
| (Lorenzoni et al. 2019) (57) | **2. Analysis:**  Predictive | **VCM2**. Discovery and experimentation  **VCM3**. Segmentation | **VT1** - Decision making:  C - Risk detection  **VT2** - Innovation:  C - Precision medicine  **VT3** - Performance:  C - Patient flow | **B1. Operational:**  Quality improvement:  *Clinical decision*  **B2. Organizational:**  Change work patterns | **C1. Generation of valuable knowledge:**  Access to data  Techno related challenges |
| (Madsen, Ladelund, et Linneberg 2014) (58) | **2. Analysis:**  Analytical | **VCM1**. Process and outcomes transparency  **VCM2**. Discovery and experimentation | **VT2** - Innovation:  M - Adapt strategies  **VT3** - Performance:  M - Operations management | **B1. Operational:**  Productivity gains  Cost reduction:  *Other*  **B3. Managerial**  Resource management | **C1. Generation of valuable knowledge:**  Access to data  Methodological challenges  **C3. Challenges for hospitals to invest in BDA strategies**:  Management challenges |
| (Mahajan et al. 2019) (59) | **1. Acquisition:**  Traceability  Interoperability  **2. Analysis:**  Analytical  **3. Interpretation:**  Decision support | **VCM1**. Process and outcomes transparency  **VCM2**. Discovery and experimentation  **VCM4**. Predict | **VT1** - Decision making:  M - Assess activities | **B1. Operational:**  Quality improvement:  *Clinical outcomes*  **B2. Organizational:**  Change work patterns  Communication and collaboration  **B3. Managerial**  Decision-making and planning  Performance improvement  **B4. Strategic:**  Innovation |  |
| (Martin et al. 2019) (60) | **2. Analysis:**  Analytical  **3. Interpretation:**  Decision support | **VCM2**. Discovery and experimentation  **VCM5**. Monitoring | **VT2** - Innovation:  C - Preventative medicine  **VT3** - Performance:  C - Patient flow | **B1. Operational:**  Cost reduction:  *Reduce readmissions*  Quality improvement: *Clinical decision*  **B2. Organizational:**  Change work patterns  Communication and collaboration | **C2. Transforming knowledge into actions:**  Acceptance |
| (McNair 2015) (61) | **1. Acquisition:**  Traceability  **2. Analysis:**  Predictive | **VCM2**. Discovery and experimentation  **VCM4**. Predict | **VT1** - Decision making:  M - Resource allocation | **B1. Operational:**  Cost reduction:  *Other*  Quality improvement:  *Clinical outcomes*  Service improvement:  *For HCPs*  **B2. Organizational:**  Morale and satisfaction  **B3. Managerial**  Resource management  **B4. Strategic:**  Differentiation  Positioning  Resources |  |
| (Moon et al. 2019) (62) | **1. Acquisition:**  Traceability  **2. Analysis:**  Analytical | **VCM1**. Process and outcomes transparency | **VT1** - Decision making  C - Diagnostic | **B1. Operational:**  Productivity gains | **C1. Generation of valuable knowledge:**  Access to data  Lack of talents  **C2. Transforming knowledge into actions:**  Complexity |
| (Moss et al. 2016) (63) | **1. Acquisition:**  Traceability  **2. Analysis:**  Analytical | **VCM2**. Discovery and experimentation | **VT1** - Decision making:  C - Diagnostic | **B1. Operational:**  Quality improvement:  *Clinical decision*  **B2. Organizational:**  Support learning and skills development | **C1. Generation of valuable knowledge:**  Access to data  Data quality  Lack of talents |
| (Navarro et al. 2018) (64) | **1. Acquisition:**  Traceability  **2. Analysis:**  Predictive | **VCM2**. Discovery and experimentation  **VCM3**. Segmentation | **VT2** - Innovation:  C - Precision medicine  M - Adapt strategies  **VT3** - Performance:  C - Patient flow | **B2. Organizational:**  Change work patterns  Communication and collaboration  **B3. Managerial**  Resource management  **B4. Strategic:**  Innovation | **C1. Generation of valuable knowledge:**  Techno related challenges |
| (Ouchi et al. 2018) (65) | **2. Analysis:**  Predictive | **VCM2**. Discovery and experimentation  **VCM3**. Segmentation | **VT2** - Innovation:  C - Preventative medicine | **B1. Operational:**  Productivity gains  Quality improvement:  *Clinical decision*  **B2. Organizational:**  Communication and collaboration | **C1. Generation of valuable knowledge:**  Access to data  Data quality  Techno related challenges  Methodological challenges  Lack of talents  **C2. Transforming knowledge into actions:**  Acceptance |
| (Petrozziello et al. 2018) (66) | **2. Analysis:**  Predictive  **3. Interpretation:**  Decision support | **VCM2**. Discovery and experimentation  **VCM3**. Segmentation  **VCM5**. Monitoring | **VT1** - Decision making:  C – Diagnostic  **VT2** - Innovation:  C - Precision medicine | **B1. Operational:**  Quality improvement:  *Clinical decision*  Service improvement:  *For patients*  **B3. Managerial**  Performance improvement | **C1. Generation of valuable knowledge:**  Access to data |
| (Pinsky et Dubrawski 2014) (67) | **1. Acquisition:**  Interoperability  **2. Analysis:**  Predictive  **3. Interpretation:**  Decision support | **VCM2**. Discovery and experimentation  **VCM3**. Segmentation  **VCM5**. Monitoring | **VT1** - Decision making:  C - Risk detection  **VT2** - Innovation:  C - Precision medicine | **B1. Operational:**  Quality improvement:  *Clinical outcomes*  **B2. Organizational:**  Support learning and skills development | **C1. Generation of valuable knowledge:**  Techno related challenges  Lack of talents  **C3. Challenges for hospitals to invest in BDA strategies:**  Economic challenges |
| (Pottenger et al. 2016) (68) | **1. Acquisition:**  Traceability  Interoperability  **2. Analysis:**  Analytical  **3. Interpretation:**  Decision support | **VCM1**. Process and outcomes transparency  **VCM2**. Discovery and experimentation | **VT1** - Decision making:  M - Resource allocation | **B1. Operational:**  Service improvement:  *For patients*  **B2. Organizational:**  Support learning and skills development  Communication and collaboration  **B3. Managerial**  Performance improvement | **C2. Transforming knowledge into actions:**  Complexity  Acceptance  **C3. Challenges for hospitals to invest in BDA strategies**:  Management challenges |
| (Raita et al. 2019) (69) | **2. Analysis:**  Predictive | **VCM2**. Discovery and experimentation  **VCM3**. Segmentation  **VCM4**. Predict | **VT1** - Decision making:  C – Diagnostic  M - Resource allocation  **VT2** - Innovation:  C - Precision medicine  **VT3** - Performance:  C - Patient flow | **B1. Operational:**  Productivity gains  Cost reduction:  *Unnecessary care*  Quality improvement:  *Clinical outcomes*  **B3. Managerial**  Resource management | **C1. Generation of valuable knowledge:**  Access to data  Techno related challenges |
| (Rajkomar et al. 2018) (70) | **2. Analysis:**  Predictive | **VCM2**. Discovery and experimentation | **VT1** - Decision making:  C - Risk detection  **VT3** - Performance:  R - Research performance | **B1. Operational:**  Productivity gains  **B3. Managerial**  Decision-making and planning | **C1. Generation of valuable knowledge:**  Data quality  Techno related challenges |
| (Ramkumar, Haeberle, et al. 2019) (71) | **2. Analysis:**  Analytical  Predictive | **VCM2**. Discovery and experimentation  **VCM3**. Segmentation  **VCM4**. Predict  **VCM5**. Monitoring | **VT1** - Decision making:  C - Risk detection  M - Assess activities  **VT2** - Innovation:  C - Precision medicine  C - Preventative medicine  **VT3** - Performance:  C - Patient flow  M - Operations management | **B1. Operational:**  Productivity gains  Quality improvement:  *Clinical decision*  *Clinical outcomes*  Service improvement:  *For patients*  *For HCPs*  **B2. Organizational:**  Change work patterns  **B3. Managerial**  Decision-making and planning  Performance improvement  **B4. Strategic:**  Differentiation  Innovation | **C1. Generation of valuable knowledge:**  Data quality  Techno related challenges  **C2. Transforming knowledge into actions:**  Complexity  Acceptance  **C3. Challenges for hospitals to invest in BDA strategies:**  Economic challenges |
| (Ramkumar, Navarro, et al. 2019) (72) | **2. Analysis:**  Predictive | **VCM2**. Discovery and experimentation  **VCM3**. Segmentation | **VT2** - Innovation:  C - Precision medicine  M - Adapt strategies | **B1. Operational:**  Revenue growth  Cost reduction:  *Unnecessary care*  Service improvement:  *For patients*  **B4. Strategic:**  Differentiation  Innovation | **C1. Generation of valuable knowledge:**  Techno related challenges  Methodological challenges  **C3. Challenges for hospitals to invest in BDA strategies:**  Management challenges |
| (Ratliff et al. 2016) (73) | **1. Acquisition:**  Traceability  **2. Analysis:**  Predictive | **VCM1**. Process and outcomes transparency  **VCM2**. Discovery and experimentation  **VCM3**. Segmentation | **VT1** - Decision making:  C - Risk detection  **VT2** - Innovation:  C - Preventative medicine | **B1. Operational:**  Service improvement:  *For patients*  **B2. Organizational:**  Support learning and skills development  **B3. Managerial**  Decision-making and planning  **B4. Strategic:**  Innovation | **C1. Generation of valuable knowledge:**  Data quality  Techno related challenges  Lack of talents |
| (Ratwani et Fong 2015) (74) | **2. Analysis:**  Analytical  **3. Interpretation:**  Decision support | **VCM1**. Process and outcomes transparency  **VCM2**. Discovery and experimentation | **VT1** - Decision making:  M - Assess activities | **B1. Operational**:  Productivity gains  **B2. Organizational:**  Change work patterns | **C1. Generation of valuable knowledge:**  Lack of talents |
| (Raza et al. 2019) (75) | **2. Analysis:**  Predictive | **VCM2**. Discovery and experimentation  **VCM3**. Segmentation | **VT1** - Decision making:  C - Risk detection  **VT2** - Innovation:  C - Precision medicine | **B1. Operational:**  Quality improvement:  *Clinical decision*  *Clinical outcomes* |  |
| (Robinson et al. 2017) (76) | **1. Acquisition:**  Traceability  Interoperability  **2. Analysis:**  Analytical  **3. Interpretation:**  Decision support | **VCM2**. Discovery and experimentation  **VCM3**. Segmentation | **VT1** – Decision making  M - Assess activities  VT2 - Innovation:  C - Preventative medicine  **VT3** - Performance:  C - Patient flow | **B1. Operational:**  Cost reduction:  *Other*  **B2. Organizational:**  Change work patterns | **C1. Generation of valuable knowledge:**  Access to data  Data quality  Techno related challenges  **C2. Transforming knowledge into actions:**  Complexity  Acceptance |
| (Rocchio 2016) (77) | **2. Analysis:**  Analytical  **3. Interpretation:**  Decision support | **VCM1**. Process and outcomes transparency  **VCM2**. Discovery and experimentation  **VCM5**. Monitoring | **VT1** - Decision making:  M - Assess activities  **VT2** - Innovation:  C - Precision medicine | **B1. Operational:**  Cost reduction:  *Other*  **B2. Organizational:**  Communication and collaboration  **B3. Managerial**  Decision-making and planning  Performance improvement | **C1. Generation of valuable knowledge:**  Data quality  Lack of talents  **C2. Transforming knowledge into actions:**  Acceptance |
| (Ruiz-Cordell, Joubin, et Haimowitz 2016) (78) | **1. Acquisition:**  Interoperability  **2. Analysis:**  Predictive | **VCM2**. Discovery and experimentation | **VT1** - Decision making:  M - Assess activities | **B1. Operational:**  Service improvement:  *For HCPs*  **B2. Organizational:**  Support learning and skills development  **B3. Managerial**  Decision-making and planning  Performance improvement |  |
| (Ruminski et al. 2019) (79) | **2. Analysis:**  Predictive  **3. Interpretation:**  Decision support | **VCM2**. Discovery and experimentation  **VCM3**. Segmentation  **VCM5**. Monitoring | **VT1** - Decision making:  C - Risk detection  **VT2** - Innovation:  C - Precision medicine | **B1. Operational:**  Quality improvement:  *Clinical decision*  *Clinical outcomes*  **B2. Organizational**:  Change work patterns  Communication and collaboration | **C1. Generation of valuable knowledge:**  Data quality  Techno related challenges  **C2. Transforming knowledge into actions:**  Limited awareness  Acceptance |
| (Ryan, Hendler, et Bennett 2015) (80) | **1. Acquisition:**  Traceability  **2. Analysis:**  Analytical | **VCM1**. Process and outcomes transparency  **VCM2**. Discovery and experimentation  **VCM3**. Segmentation | **VT3** - Performance:  C - Patient flow | **B1. Operational:**  Cost reduction:  *Reduce readmissions*  Quality improvement:  *Clinical decision*  **B2. Organizational:**  Change work patterns | **C1. Generation of valuable knowledge:**  Access to data  Methodological challenges  **C2. Transforming knowledge into actions:**  Complexity |
| (Sandquist et Tegtmeyer 2018) (81) | **2. Analysis:**  Predictive  **3. Interpretation:**  Decision support | **VCM2**. Discovery and experimentation  **VCM4**. Predict | **VT1** - Decision making:  C - Risk detection  **VT2** - Innovation:  C - Precision medicine | **B1. Operational**:  Cost reduction:  *Other*  Quality improvement:  *Clinical decision*  Service improvement:  *For HCPs* | **C1. Generation of valuable knowledge:**  Access to data  Techno related challenges  Methodological challenges  **C2. Transforming knowledge into actions:**  Acceptance |
| (Schuetz et Larson 2019) (82) | **2. Analysis:**  Predictive | **VCM5**. Monitoring | **VT1** - Decision making:  M - Resource allocation | **B1. Operational:**  Productivity gains  Cost reduction:  *Other*  **B2. Organizational:**  Support learning and skills development  Morale and satisfaction  **B3. Managerial**  Resource management  Decision-making and planning  Performance improvement  **B4. Strategic:**  Positioning  Resources | **C2. Transforming knowledge into actions:**  Complexity |
| (Shi et al. 2018) (83) | **1. Acquisition:**  Traceability  Interoperability  **2. Analysis:**  Analytical  **3. Interpretation:**  Decision support | **VCM2**. Discovery and experimentation  **VCM5**. Monitoring | **VT1** - Decision making:  C – Diagnostic  C - Risk detection  M - Resource allocation / | **B1. Operational:**  Productivity gains  Service improvement:  *For HCPs*  **B2. Organizational:**  Change work patterns  Morale and satisfaction  **B3. Managerial**  Performance improvement | **C1. Generation of valuable knowledge:**  Techno related challenges  **C2. Transforming knowledge into actions:**  Acceptance |
| (Silahtaroğlu et Yılmaztürk 2019) (84) | **1. Acquisition:**  Interoperability  **2. Analysis:**  Analytical  Predictive | **VCM2**. Discovery and experimentation  **VCM3**. Segmentation  **VCM4**. Predict | **VT1** - Decision making:  C - Diagnostic | **B1. Operational:**  Cost reduction:  *Unnecessary care*  Quality improvement:  *Clinical decision*  Service improvement:  *For patients*  **B2. Organizational**:  Change work patterns | **C1. Generation of valuable knowledge:**  Data quality |
| (Singh et al. 2017) (85) | **1. Acquisition:**  Traceability  Interoperability  **2. Analysis:**  Analytical  **3. Interpretation:**  Decision support | **VCM1**. Process and outcomes transparency  **VCM2**. Discovery and experimentation  **VCM5**. Monitoring | **VT2** - Innovation:  C - Precision medicine | **B1. Operational:**  Productivity gains  Quality improvement:  *Clinical decision*  *Clinical outcomes*  **B2. Organizational:**  Support learning and skills development  Change work patterns | **C1. Generation of valuable knowledge:**  Techno related challenges  **C2. Transforming knowledge into actions:**  Complexity  Acceptance |
| (Song et al. 2019) (86) | **1. Acquisition:**  Interoperability  **2. Analysis:**  Predictive | **VCM2**. Discovery and experimentation  **VCM4**. Predict | **VT3** - Performance:  M - Operations management | **B1. Operational:**  Cost reduction:  *Other*  Quality improvement:  Clinical outcomes  **B2. Organizational:**  Change work patterns  **B3. Managerial:**  Resource management |  |
| (Spangenberg, Wilke, et Franczyk 2017) (87) | **1. Acquisition:**  Interoperability  **2. Analysis:**  Predictive  **3. Interpretation:**  Decision support | **VCM1**. Process and outcomes transparency  **VCM2**. Discovery and experimentation  **VCM5**. Monitoring | **VT1** - Decision making:  M - Resource allocation | **B1. Operational:**  Productivity gains  Service improvement:  *For HCPs*  **B3. Managerial**  Resource management | **C1. Generation of valuable knowledge:**  Access to data  Data quality  Techno related challenges  Methodological challenges  **C2. Transforming knowledge into actions:**  Complexity  Acceptance |
| (Stadler et al. 2016) (88) | **1. Acquisition:**  Interoperability  **2. Analysis:**  Analytical  **3. Interpretation:**  Decision support | **VCM2**. Discovery and experimentation  **VCM5**. Monitoring | VT1 - Decision making:  M - Assess activities | **B1. Operational:**  Productivity gains  **B2. Organizational:**  Change work patterns  Communication and collaboration  **B3. Managerial**  Resource management  Performance improvement  **B4. Strategic:**  Differentiation | **C1. Generation of valuable knowledge:**  Access to data  Data quality  **C2. Transforming knowledge into actions:**  Complexity  Acceptance  **C3. Challenges for hospitals to invest in BDA strategies:**  Economic challenges |
| (Taylor et al. 2016) (89) | **2. Analysis:**  Predictive | **VCM2**. Discovery and experimentation  **VCM3**. Segmentation | **VT1** - Decision making:  C - Risk detection  **VT2** - Innovation:  C - Precision medicine | **B1. Operational:**  Cost reduction:  *Unnecessary care*  Quality improvement:  *Clinical decision*  *Clinical outcomes*  **B2. Organizational:**  Change work patterns  Communication and collaboration | **C1. Generation of valuable knowledge:**  Access to data  Techno related challenges  Methodological challenges  **C2. Transforming knowledge into actions:**  Acceptance |
| (Yang et al. 2019) (90) | **1. Acquisition:**  Traceability  **2. Analysis:**  Analytical  Predictive | **VCM2**. Discovery and experimentation  **VCM3**. Segmentation | **VT1** - Decision making:  C – Diagnostic  **VT2** - Innovation:  C - Precision medicine | **B1. Operational:**  Quality improvement:  *Clinical decision*  **B2. Organizational:**  Support learning and skills development | **C1. Generation of valuable knowledge:**  Access to data |
| (Ye et al. 2019) (91) | **1. Acquisition:**  Traceability  **2. Analysis:**  Analytical | **VCM2**. Discovery and experimentation  **VCM3**. Segmentation | **VT1** - Decision making:  M - Resource allocation  **VT2** - Innovation:  M - Adapt strategies | **B1**. **Operational:**  Service improvement:  *For patients*  **B2. Organizational:**  Communication and collaboration  **B3**. **Managerial**  Decision-making and planning  Performance improvement  **B4**. **Strategic:**  Differentiation  Positioning  Influence | **C1. Generation of valuable knowledge:**  Techno related challenges  Methodological challenges  **C3. Challenges for hospitals to invest in BDA strategies:**  Economic challenges |
| (Zhang, Wang, et Pauleen 2017) (92) | **2. Analysis:**  Analytical  **3. Interpretation:**  Decision support | **VCM1**. Process and outcomes transparency  **VCM2**. Discovery and experimentation | **VT1** - Decision making:  M - Assess activities | **B1. Operational:**  Cost reduction:  *Unnecessary care*  Quality improvement:  *Clinical outcomes*  **B2. Organizational:**  Change work patterns  **B3. Managerial**  Decision-making and planning |  |
| (Zhu et al. 2015) (93) | **1. Acquisition:**  Traceability  **2. Analysis:**  Predictive | **VCM2**. Discovery and experimentation  **VCM3**. Segmentation  **VCM4**. Predict | **VT1** - Decision making:  C - Risk detection  **VT2** - Innovation:  C - Preventative medicine | **B1. Operational:**  Cost reduction:  *Reduce readmissions*  Quality improvement:  *Clinical decision*  **B2**. **Organizational:**  Support learning and skills development  Change work patterns | **C1. Generation of valuable knowledge:**  Access to data  Techno related challenges  Methodological challenges  **C2. Transforming knowledge into actions**:  Limited awareness  Acceptance |
| (Zolbanin et Delen 2018) (94) | **2. Analysis:**  Predictive | **VCM2**. Discovery and experimentation  **VCM3**. Segmentation | **VT2** - Innovation:  C - Preventative medicine  **VT3** - Performance:  C - Patient flow | **B2. Organizational:** Change work patterns  Communication and collaboration | **C1. Generation of valuable knowledge:**  Access to data  Techno related challenges |

**Table 3** – Summary of study articles

| ***Content*** | | ***References*** |
| --- | --- | --- |
| ***1/ Big data capabilities*** | |  |
|  | **Data acquisition: 54** |  |
|  | Traceability: 43 | (4,8–10,12–16,18,19,22,23,25,28,30–32,35,39,43–45,47,50,52–55,59,61–64,68,73,76,80,83,85,90,91,93) |
|  | Interoperability: 28 | (7,9,13,18,19,21,24,27,29,30,35,43,45,50,52,54,55,59,67,68,76,78,83–88) |
|  | **Data analysis: 94** |  |
|  | Analytical: 58 | (3,4,6–9,11–19,22,23,26,27,29–32,34,35,47,49–54,58–60,62,63,68,71,74,76,77,80,83–85,88,88,90–92) |
|  | Predictive: 50 | (1–3,5,10–12,17,18,20,21,24,25,28,30,32,33,36–38,40,42,44,45,49,55–57,61,64–67,69–73,75,78,79,81,82,84,86,87,89,90,93,94) |
|  | **Decision support: 44** | (5–9,12–15,17,18,21,22,26,27,29,32,35–37,40,43,45,47,48,51,53,56,59,60,66–68,74,76,77,81,83,83,85,87,88,92) |
| ***2/ Value creation mechanisms*** | |  |
|  | **VCM1. Process and outcomes transparency:** 42 |  |
|  | Process:17 | (7,9,12–14,25,29,32,39,41,47,53,68,77,80,87,92) |
|  | Outcomes: 25 | (3,4,6,8,14–16,18,19,22–24,26,27,30,40,44–46,49,58,59,62,73,74) |
|  | **VCM2. Discovery and experimentation: 83** | (1–10,12,12–14,16–27,29–31,33–35,35,36,38–43,45–47,49–61,63–71,74–81,83–86,88,90–94) |
|  | **VCM3. Segmentation: 46** | (1,2,45–49,55–57,64–67,69,71–73,75,76,79,80,84,89–91,93,94) |
|  | **VCM4. Predict: 19** | (10,17,18,21,24,32,35–37,51,59,61,69,71,81,84,86,93) |
|  | **VCM5. Monitoring: 25** | (5,6,8,11,15,28,34,36,40,45,47,48,53,60,66,67,71,77,79,82,83,85,87,88) |
| ***3/ Value targets*** | |  |
|  | **VT1 – Decision making: 62** |  |
|  | Diagnostic – C: 12 | (11,20,37,38,54,62,63,66,69,83,84,90) |
|  | Risk detection – C: 22 | (1–3,5,21,24,28,30,42,46,50,57,67,70,71,73,75,79,81,83,89,93) |
|  | Assessing hospital activities – M: 20 | (4,6,9,14–17,27,33,34,44,47,59,71,74,76–78,88,92) |
|  | Resource allocation – M: 14 | (5,13,27,32,36,41,52,61,68,69,82,83,87,91) |
|  | Hypothesis setting – R: 2 | (34,41) |
|  | **VT2 – Innovation: 54** |  |
|  | Precision medicine – C: 27 | (3,5,14,20,21,24,30,38,40,44,48,49,56,57,64,66,67,69,71,72,75,77,79,81,85,89,90) |
|  | Preventative medicine – C: 22 | (2,6,8,10,14,15,18,23,26,28,35,40,42,53,55,60,65,71,73,76,93,94) |
|  | Adapt strategies – M: 10 | (14,15,17,22,39,45,58,64,72,91) |
|  | New research tools – R: 2 | (34,41) |
|  | **VT3 – Performance: 36** |  |
|  | Patient flow – C: 25 | (6,9,11,12,14,25,31,34–37,39,40,46,47,49,55,60,64,69,71,76,80,94) |
|  | Operations management – A: 7 | (7,13,32,51,58,71,86) |
|  | Research performance – R: 6 | (19,29,43,50,54,70) |
| ***4/ Benefits*** | |  |
|  | **Operational: 83** |  |
|  | Revenue growth: 2 | (35,72) |
|  | Productivity gains: 26 | (4,5,12,17,18,21,25,26,29,33,35,37,39,43,47,50,58,62,65,69,71,82,83,85,87,88) |
|  | Cost reduction / savings 38 |  |
|  | *Reduce unnecessary care: 13* | (3,11,17,24,28,38,46,56,69,72,84,89,92) |
|  | *Reduce readmissions: 6* | (30,35,55,60,80,93) |
|  | *Other: 21* | (25,28,32,33,33,38,40,42,45,51,58,61,76,77,81,82,86) |
|  | Quality improvement: 50 |  |
|  | *Clinical decisions: 34* | (1–3,5,10,11,14,17,18,20,23,24,30,36,38,40,43,53,57,60,63,65,66,71,75,79–81,84,85,89,90,93) |
|  | *Clinical outcomes: 27* | (2,3,13,14,20,21,29,30,32,38,39,45,47,48,52,56,59,61,67,69,71,75,79,85,86,89,92) |
|  | Service improvement: 27 |  |
|  | *For patients: 12* | (4,25,35,36,46,66,68,71–73,84,91) |
|  | *For healthcare professionals: 17* | (8,11,14,21,29,35,37,39,40,51,56,61,71,78,81,83,87) |
|  | **Organizational: 62** |  |
|  | Support learning and skills development: 21 | (2,8,10,13,14,17,18,29,33,34,38,45,67,68,70,73,78,82,85,90,93) |
|  | Change work patterns: 41 |  |
|  | *Within team: 28* | (28,30,31,39,41,42,44,47–49,55–57,60,64,64,71,76,79,80,86,88,89,92–94) |
|  | *Across functions: 13* | (3,9,11,13,23,33,35,45,59,74,83–85) |
|  | *With other HCOs: 3* | (35,84,85) |
|  | Improve cross-functional communication and collaboration: 17 | (6,33,39,41,43,45,59,60,64,65,68,77,79,88,89,91,94) |
|  | Increase employee morale and satisfaction: 3 | (61,82,83) |
|  | **Managerial: 52** |  |
|  | Better resource management: 31 | (2,6,7,13,16,17,19,22,24,27,28,32,33,36,39,41,44,45,47,48,51,52,55,58,61,64,69,82,86–88) |
|  | Improved decision-making and planning: 18 | (7,9,13,16,22,27,33,34,49,59,70,71,73,77,78,82,91,92) |
|  | Performance improvement: 22 | (4,9,15,17–19,23,25,27,43,45,59,66,68,71,77,78,82,83,88,91,91) |
|  | **Strategic: 26** |  |
|  | Use BDA as a differentiator: 12 | (14,14,15,17,17,18,18,33,33,43,43,44,61,61,71,71,72,72,88,88,91) |
|  | Support for business growth (activities, services): 5 | (14,35,39,41,43) |
|  | Support for business innovations (services, process): 9 | (45,49,59,64,71–73) |
|  | Positioning: 7 |  |
|  | *Attract resources: 4* | (14,45,61,82) |
|  | *Gain influence: 3* | (23,44,91) |
| **5/ Challenges** | |  |
|  | **C1. Generation of valuable knowledge 79** |  |
|  | Data related challenges: |  |
|  | Access: 48 | (2–5,8–11,15,16,18,19,21–27,30,32,34,38,43–45,47,50–52,54,55,57,58,62,63,65,66,69,76,80,86,88,89,93,94) |
|  | Quality: 33 | (1,3,7,10,16,18,21,23,26,29,32,33,36,37,40,42,45,49,50,52,54,55,65,70,71,73,76,77,79,84,87,88) |
|  | Techno related challenges: 49 | (3,5,7–13,15,19,25,27–30,34–36,38,39,43,45,47–50,52,53,57,64,65,67,69–73,76,79,81,83,85,87,89,91,93,94) |
|  | Methodological challenges: 16 | (1,18,19,40,48–50,58,65,72,80,81,87,89,91,93) |
|  | Lack of talents: 17 | (15,17,21,22,33,40,41,45,53,62,63,65,67,72–74,77) |
|  | **C2. Transforming knowledge into actions** 42 |  |
|  | Limited awareness: 4 | (9,10,79,93) |
|  | Complexity: 19 | (2,9,14,21,27,35,38,41–43,56,62,68,71,76,80,82,87,88) |
|  | Acceptance: 33 | (9,13,14,17,19,21,26,27,34,38–40,48,53,54,60,65,68,71,76,77,79,81,83,85,87–89,93) |
|  | **C3. Challenges for hospitals to invest in BDA strategies: 21** |  |
|  | Economic challenges: 13 | (10,17,21,23,29,40,42,43,45,67,71,88,91) |
|  | Management challenges: 11 | (14,17,18,35,43–45,52,58,68,72) |

**Supplementary Table 4.** Distribution by expected and measured benefits

| **Items** | **N expected**  **(N realized)** | **References** | **Context** |
| --- | --- | --- | --- |
| **Operational** | **83 (9)** |  |  |
| Revenue growth | 2 (1) | Hewner, Sullivan et Yu (2018) | Generation of additional revenues from outpatient visits for patients benefiting from transitional care after being identified as being at-risk by classification algorithms. |
| Productivity gains | 26 (2) | Schuetz et Larson (2019) | The use of predictive analytics to support nurse staffing and scheduling help save up to 50% of time dedicated by managers on these activities while improving scheduling outcomes. |
| Cost reduction / savings | 38 |  |  |
| Reduce unnecessary care | 13 (4) | Raita et al. (2019) | Use machine learning to support emergency department triage and reduce over-triaging to better allocate resources towards patients in needs. |
| Reduce readmissions | 6 (2) | Golas et al. (2018) | Reduce readmissions and healthcare costs with targeted interventions following hospitalization for heart failure by better targeting high-risks patients at discharge using risk prediction model developed using deep unified networks. |
| Other | 21 | Song (2017) | Reduce costs due to drugs overstocking or shortages by using a deep neural network to predict morbidities of acute gastrointestinal infections and a heuristic optimization algorithm to improve drug procurement planning and use. |
| Quality improvement | 50 |  |  |
| Clinical decisions | 34 (1) | Al’Aref et al. (2019) | Identify optimal revascularization strategy for patients requiring coronary intervention by utilizing traditional and advanced machine learning to determine the most significant predictors (association of risk factors) of in-hospital mortality. |
| Clinical outcomes | 27 (3) | Singh (2017) | Reduce mortality, complication and morbidity of time sensitive conditions in patients at the emergency department by using descriptive, diagnostic, prescriptive analytics methods to identify areas of deficiency and potential improvement emergency department performance. |
| Service improvement | 22 |  |  |
| For patients | 12 (2) | Ye et al. (2019) | Use machine learning approaches to derive similarity matches and sentiment analysis to generate physician rankings and help patients better chose appropriate doctors. |
| For healthcare professionals | 17 (2) | Spangenberg, Wilke, et Franczyk (2017) | Release healthcare professionals from observing current status of remaining intervention times in operating rooms by developing predictive regression algorithms. |
| **Organizational** | **62 (7)** |  |  |
| Support learning and skills development | 21 | Yang et al. (2019) | Support learning of healthcare professionals by leveraging predictive techniques to identify patients at risk and engage them in changing their practices by adapting protocols to newly revealed patient needs. |
| Change work patterns 1 | 41 |  |  |
| Within team | 28 (3) | Martin (2019) | Leveraging descriptive techniques to better understand potentially preventable hospitalization trajectories and generate alerts to healthcare professionals and enable anticipatory care approaches within the emergency department. |
| Across functions | 13 (1) | Cheng et Kuo (2016) | RFID analytics to explore data generated by tags on staffs and assets and provide intelligence for hospital ward management to define new workflows that can enhance patient safety, increase operational efficiency and mitigate risks of infectious diseases. |
| With other HCOs | 3 | Singh (2017) | Use predictive techniques to identify patients at risk in real time and help hospital to fill their skill gaps by referring to other institutions when expertise is needed. |
| Improve cross-functional communication and collaboration | 17 (1) | Rocchio (2016) | Develop perioperative dashboards to facilitate communication and interactions between perioperative leaders, surgeons and perioperative staff members to analyze healthcare data and discuss solutions for cost-savings. |
| Increase employee morale and satisfaction | 3 (2) | Schuetz et Larson (2019) | Build on the performance of analytics to improve staffing and scheduling to introduce new attractive open shifts incentives that can contribute to improving staff satisfaction. |
| **Managerial** | **52 (6)** |  |  |
| Better resource management | 31 (3) | Leary et al. (2016) | Identify relationships between staffing levels and clinical outcomes to support the development of mathematical model that will enable managers to optimize nurse staffing. |
| Improved decision-making and planning | 18 (2) | Mahajan et al. (2019) | Development of data analytics supported dashboards assessing 26 quality measures to help managers and clinical staff make informed decision on where to start making changes and how to explore the consequences of potential actions. |
| Performance improvement | 22 (1) | Foster et al. (2018) | Leverage big data to propose data-driven metrics to objectively evaluate physicians across facilities and enable administrators to better manage and incentivize physicians and provide direction for performance improvement. |
| **Strategic** | **26 (4)** |  |  |
| Use BDA as a differentiator | 12 (2) | Ramkumar et al. (2019) | Use machine learning to develop patient specific payment mode that enable hospitals to reduce patient selections and offer improved access to care. |
| Support for business growth (activities, services) | 5 (1) | Ibanez-Sanchez et al. (2019) | Apply process mining tools to support healthcare professionals in the development of value-based approaches to care delivery. |
| Support for business innovations (services, process) | 9 | Navaro et al. (2018) | Machine learning algorithms enable prediction of length of stay and costs to support value-based applications including new risk-based patient-specific payment models. |
| Positioning | 7 |  |  |
| Attract resources | 4 | McNair (2015)r | Optimized staffing and scheduling may improve nurse retention in highly competitive markets. |
| Gain influence | 3 (1) | Dreyfus et al. (2018) | Use descriptive techniques to investigate hospital-acquired pressure injuries risk factors and outcomes and inform providers and policy-makers on best practices for prevention and influence how risk should be assessed. |

**Bibliography of the appendix section**

1. Al’Aref SJ, Singh G, van Rosendael AR, Kolli KK, Ma X, Maliakal G, et al. Determinants of In‐Hospital Mortality After Percutaneous Coronary Intervention: A Machine Learning Approach. JAHA [Internet]. 5 mars 2019 [cité 5 juin 2020];8(5). Disponible sur: https://www.ahajournals.org/doi/10.1161/JAHA.118.011160

2. Alnsour Y, Hadidi R, Singh N. Using Data Analytics to Predict Hospital Mortality in Sepsis Patients: International Journal of Healthcare Information Systems and Informatics. juill 2019;14(3):40‑57.

3. An S, Malhotra K, Dilley C, Han-Burgess E, Valdez JN, Robertson J, et al. Predicting drug-resistant epilepsy — A machine learning approach based on administrative claims data. Epilepsy & Behavior. déc 2018;89:118‑25.

4. Anderson JE, Chang DC. Using Electronic Health Records for Surgical Quality Improvement in the Era of Big Data. JAMA Surg. 1 janv 2015;150(1):24.

5. Avati A, Jung K, Harman S, Downing L, Ng A, Shah NH. Improving Palliative Care with Deep Learning. 2018;10.

6. Baechle C, Agarwal A. A framework for the estimation and reduction of hospital readmission penalties using predictive analytics. J Big Data. déc 2017;4(1):37.

7. Baghapour MA, Shooshtarian MR, Javaheri MR, Dehghanifard S, Sefidkar R, Nobandegani AF. A computer-based approach for data analyzing in hospital’s health-care waste management sector by developing an index using consensus-based fuzzy multi-criteria group decision-making models. International Journal of Medical Informatics. oct 2018;118:5‑15.

8. Bouzillé G, Morival C, Westerlynck R, Lemordant P, Chazard E, Lecorre P, et al. An Automated Detection System of Drug-Drug Interactions from Electronic Patient Records Using Big Data Analytics. :5.

9. Bygstad B, Øvrelid E, Lie T, Bergquist M. Developing and Organizing an Analytics Capability for Patient Flow in a General Hospital. Inf Syst Front [Internet]. 4 mai 2019 [cité 22 nov 2019]; Disponible sur: http://link.springer.com/10.1007/s10796-019-09920-2

10. Calcaterra SL, Scarbro S, Hull ML, Forber AD, Binswanger IA, Colborn KL. Prediction of Future Chronic Opioid Use Among Hospitalized Patients. J GEN INTERN MED. juin 2018;33(6):898‑905.

11. Chen J, Li K, Tang Z, Bilal K, Li K. A Parallel Patient Treatment Time Prediction Algorithm and Its Applications in Hospital Queuing-Recommendation in a Big Data Environment. IEEE Access. 2016;4:1767‑83.

12. Chen Z, Bird VY, Ruchi R, Segal MS, Bian J, Khan SR, et al. Development of a personalized diagnostic model for kidney stone disease tailored to acute care by integrating large clinical, demographics and laboratory data: the diagnostic acute care algorithm - kidney stones (DACA-KS). BMC Med Inform Decis Mak. déc 2018;18(1):72.

13. Cheng C-H, Kuo Y-H. RFID analytics for hospital ward management. Flex Serv Manuf J. déc 2016;28(4):593‑616.

14. Clarke R, Hackbarth AS, Saigal C, Skootsky SA. Building the Infrastructure for Value at UCLA: Engaging Clinicians and Developing Patient-Centric Measurement. Academic Medicine. oct 2015;90(10):1368‑72.

15. Clifford RJ, Chukwuma U, Sparks ME, Richesson D, Neumann CV, Waterman PE, et al. Semi-Automated Visualization and ANalysis of Trends: A “SAVANT” for Facilitating Antimicrobial Stewardship Using Antistaphylococcal Resistance and Consumption as a Prototype. Open Forum Infectious Diseases [Internet]. 1 avr 2018 [cité 5 juin 2020];5(4). Disponible sur: https://academic.oup.com/ofid/article/doi/10.1093/ofid/ofy066/4951839

16. Cobb AN, Eguia E, Janjua H, Kuo PC. Put Me in the Game Coach! Resident Participation in High-risk Surgery in the Era of Big Data. Journal of Surgical Research. déc 2018;232:308‑17.

17. Cresswell K, Coleman J, Smith P, Swainson C, Slee A, Sheikh A. Qualitative analysis of multi-disciplinary round-table discussions on the acceleration of benefits and data analytics through hospital electronic prescribing (ePrescribing) systems. jhi. 4 juill 2016;23(2):501.

18. Dagliati A, Sacchi L, Tibollo V, Cogni G, Teliti M, Martinez-Millana A, et al. A dashboard-based system for supporting diabetes care. Journal of the American Medical Informatics Association. 1 mai 2018;25(5):538‑47.

19. Damle R, Alavi K. The University Healthsystem Consortium clinical database: An emerging resource in colorectal surgery research. Seminars in Colon and Rectal Surgery. juin 2016;27(2):92‑5.

20. Danner OK. Physiologically-based, predictive analytics using the heart-rate-to-systolic-ratio significantly improves the timeliness and accuracy of sepsis prediction compared to SIRS. 2017;5.

21. Delahanty RJ, Kaufman D, Jones SS. Development and Evaluation of an Automated Machine Learning Algorithm for In-Hospital Mortality Risk Adjustment Among Critical Care Patients*: Critical Care Medicine. juin 2018;46(6):e481‑8.

22. Desai SS, Roberts T, Wilkerson J. Improving the Success of Strategic Management Using Big Data. World Hospitals and Health Services. 52(1):4.

23. Dreyfus J, Gayle J, Trueman P, Delhougne G, Siddiqui A. Assessment of Risk Factors Associated With Hospital-Acquired Pressure Injuries and Impact on Health Care Utilization and Cost Outcomes in US Hospitals. Am J Med Qual. juill 2018;33(4):348‑58.

24. DuBay DA, Su Z, Morinelli TA, Baliga P, Rohan V, Bian J, et al. Development and future deployment of a 5 years allograft survival model for kidney transplantation. Nephrology. août 2019;24(8):855‑62.

25. Elvira C, Ochoa A, Gonzalvez JC, Mochon F. Machine-Learning-Based No Show Prediction in Outpatient Visits. IJIMAI. 2018;4(7):29.

26. Ficheur G, Careira LF, Beuscart R, Chazard E. EpiHosp: A web-based visualization tool enabling the exploratory analysis of complications of implantable medical devices from a nationwide hospital database. :5.

27. Foster K, Penninti P, Shang J, Kekre S, Hegde GG, Venkat A. Leveraging Big Data to Balance New Key Performance Indicators in Emergency Physician Management Networks. Prod Oper Manag. oct 2018;27(10):1795‑815.

28. Genevès P, Calmant T, Layaïda N, Lepelley M, Artemova S, Bosson J-L. Scalable Machine Learning for Predicting At-Risk Profiles Upon Hospital Admission. Big Data Research. juill 2018;12:23‑34.

29. Godinho TM. ETL Framework for Real-Time Business Intelligence over Medical Imaging Repositories. J Digit Imaging. :10.

30. Golas SB, Shibahara T, Agboola S, Otaki H, Sato J, Nakae T, et al. A machine learning model to predict the risk of 30-day readmissions in patients with heart failure: a retrospective analysis of electronic medical records data. BMC Med Inform Decis Mak. déc 2018;18(1):44.

31. Gonçalves F, Pereira R, Ferreira JC, Vasconcelos JB, Melo F, Velez I. Emergency Waiting Times Data Analysis. 2018;6.

32. Guan L, Tian X, Gombar S, Zemek AJ, Krishnan G, Scott R, et al. Big data modeling to predict platelet usage and minimize wastage in a tertiary care system. MEDICAL SCIENCES. :6.

33. Halamka JD. Early Experiences With Big Data At An Academic Medical Center. Health Affairs. juill 2014;33(7):1132‑8.

34. Hendricks R. Process Mining of Incoming Patients with Sepsis. OJPHI [Internet]. 20 sept 2019 [cité 1 déc 2020];11(2). Disponible sur: https://journals.uic.edu/ojs/index.php/ojphi/article/view/10151

35. Hewner S, Sullivan SS, Yu G. Reducing Emergency Room Visits and In‐Hospitalizations by Implementing Best Practice for Transitional Care Using Innovative Technology and Big Data. Worldviews on Evidence‐Based Nursing. juin 2018;15(3):170‑7.

36. Ho AFW, To BZYS, Koh JM, Cheong KH. Forecasting Hospital Emergency Department Patient Volume Using Internet Search Data. IEEE Access. 2019;7:93387‑95.

37. Hu Y, Duan K, Zhang Y, Hossain MS, Mizanur Rahman SM, Alelaiwi A. Simultaneously aided diagnosis model for outpatient departments via healthcare big data analytics. Multimed Tools Appl. févr 2018;77(3):3729‑43.

38. Hu S-Y, Santus E, Forsyth AW, Malhotra D, Haimson J, Chatterjee NA, et al. Can machine learning improve patient selection for cardiac resynchronization therapy? Coppola G, éditeur. PLoS ONE. 3 oct 2019;14(10):e0222397.

39. Ibanez-Sanchez G, Fernandez-Llatas C, Martinez-Millana A, Celda A, Mandingorra J, Aparici-Tortajada L, et al. Toward Value-Based Healthcare through Interactive Process Mining in Emergency Rooms: The Stroke Case. IJERPH. 20 mai 2019;16(10):1783.

40. Janke AT, Overbeek DL, Kocher KE, Levy PD. Exploring the Potential of Predictive Analytics and Big Data in Emergency Care. Annals of Emergency Medicine. févr 2016;67(2):227‑36.

41. Johnson OA, Hall PS, Hulme C. NETIMIS: Dynamic Simulation of Health Economics Outcomes Using Big Data. PharmacoEconomics. févr 2016;34(2):107‑14.

42. Kang SY, Seo SW, Kim JY. Comprehensive risk factor evaluation of postoperative delirium following major surgery: clinical data warehouse analysis. Neurol Sci. avr 2019;40(4):793‑800.

43. Karanastasis E, Andronikou V, Chondrogiannis E, Tagaris A, Mourtzoukos K, Psychas A, et al. Data-empowered clinical trial design and eligible patient selection through the PONTE platform. Simulation Modelling Practice and Theory. mai 2019;93:245‑61.

44. Karnuta JM, Navarro SM, Haeberle HS, Helm JM, Kamath AF, Schaffer JL, et al. Predicting Inpatient Payments Prior to Lower Extremity Arthroplasty Using Deep Learning: Which Model Architecture Is Best? The Journal of Arthroplasty. oct 2019;34(10):2235-2241.e1.

45. Kern HP, Reagin MJ, Reese BS. Priming the Pump for Big Data at Sentara Healthcare: Frontiers of Health Services Management. 2016;32(4):15‑26.

46. Khalifa M. Reducing Emergency Department Crowding Using Health Analytics Methods: Designing AnEvidence Based Decision Algorithm. Procedia Computer Science. 2015;63:409‑16.

47. Khalifa M, Zabani I. Utilizing health analytics in improving the performance of healthcare services: A case study on a tertiary care hospital. Journal of Infection and Public Health. nov 2016;9(6):757‑65.

48. Kim SY. A deep learning model for real-time mortality prediction in critically ill children. 2019;10.

49. Krämer J, Schreyögg J, Busse R. Classification of hospital admissions into emergency and elective care: a machine learning approach. Health Care Manag Sci. mars 2019;22(1):85‑105.

50. Kreuger AL, Middelburg RA, Beckers EAM, de Vooght KMK, Zwaginga JJ, Kerkhoffs J-LH, et al. The identification of cases of major hemorrhage during hospitalization in patients with acute leukemia using routinely recorded healthcare data. Grolmusz V, éditeur. PLoS ONE. 15 août 2018;13(8):e0200655.

51. Kuhajda D. Using Survival Analysis to Evaluate Medical Equipment Battery Life. Biomedical Instrumentation & Technology. 1 mai 2016;50(3):184‑9.

52. Leary A, Cook R, Jones S, Smith J, Gough M, Maxwell E, et al. Mining routinely collected acute data to reveal non-linear relationships between nurse staffing levels and outcomes. BMJ Open. déc 2016;6(12):e011177.

53. Ledieu T. Clinical Data Analytics With Time-Related Graphical User Interfaces: Application to Pharmacovigilance. Frontiers in Pharmacology. 2018;9:9.

54. Li L, Binney LE, Carter S, Gutnikov SA, Beebe S, Bowsher‐Brown K, et al. Sensitivity of Administrative Coding in Identifying Inpatient Acute Strokes Complicating Procedures or Other Diseases in UK Hospitals. JAHA [Internet]. 16 juill 2019 [cité 8 déc 2020];8(14). Disponible sur: https://www.ahajournals.org/doi/10.1161/JAHA.119.012995

55. Lodhi MK, Ansari R, Yao Y, Keenan GM, Wilkie D, Khokhar AA. Predicting Hospital Re-Admissions from Nursing Care Data of Hospitalized Patients. In: Perner P, éditeur. Advances in Data Mining Applications and Theoretical Aspects [Internet]. Cham: Springer International Publishing; 2017 [cité 8 déc 2020]. p. 181‑93. (Lecture Notes in Computer Science; vol. 10357). Disponible sur: http://link.springer.com/10.1007/978-3-319-62701-4_14

56. Lodhi MK, Stifter J, Yao Y, Ansari R, Keenan GM, Wilkie DJ, et al. Predictive Modeling for End-of-Life Pain Outcome Using Electronic Health Records. In: Perner P, éditeur. Advances in Data Mining: Applications and Theoretical Aspects [Internet]. Cham: Springer International Publishing; 2015 [cité 7 juin 2021]. p. 56‑68. (Lecture Notes in Computer Science; vol. 9165). Disponible sur: http://link.springer.com/10.1007/978-3-319-20910-4_5

57. Lorenzoni G, Sabato SS, Lanera C, Bottigliengo D, Minto C, Ocagli H, et al. Comparison of Machine Learning Techniques for Prediction of Hospitalization in Heart Failure Patients. JCM. 24 août 2019;8(9):1298.

58. Madsen F, Ladelund S, Linneberg A. High Levels Of Bed Occupancy Associated With Increased Inpatient And Thirty-Day Hospital Mortality In Denmark. Health Affairs. juill 2014;33(7):1236‑44.

59. Mahajan A, Madhani P, Chitikeshi S, Selvaganesan P, Russell A, Mahajan P. Advanced Data Analytics for Improved Decision-Making at a Veterans Affairs Medical Center: Journal of Healthcare Management. janv 2019;64(1):54‑62.

60. Martin CM. Anticipatory Care in Potentially Preventable Hospitalizations: Making Data Sense of Complex Health Journeys. Frontiers in Public Health. 2019;6:15.

61. McNair DS. Enhancing Nursing Staffing Forecasting With Safety Stock Over Lead Time Modeling: Nursing Administration Quarterly. 2015;39(4):291‑6.

62. Moon S. Automated extraction of sudden cardiac death risk factors in hypertrophic cardiomyopathy patients by natural language processing. International Journal of Medical Informatics. 2019;7.

63. Moss TJ, Lake DE, Calland JF, Enfield KB, Delos JB, Fairchild KD, et al. Signatures of Subacute Potentially Catastrophic Illness in the ICU: Model Development and Validation*. Critical Care Medicine. sept 2016;44(9):1639‑48.

64. Navarro SM, Wang EY, Haeberle HS, Mont MA, Krebs VE, Patterson BM, et al. Machine Learning and Primary Total Knee Arthroplasty: Patient Forecasting for a Patient-Specific Payment Model. The Journal of Arthroplasty. déc 2018;33(12):3617‑23.

65. Ouchi K, Lindvall C, Chai PR, Boyer EW. Machine Learning to Predict, Detect, and Intervene Older Adults Vulnerable for Adverse Drug Events in the Emergency Department. J Med Toxicol. sept 2018;14(3):248‑52.

66. Petrozziello A, Jordanov I, Aris Papageorghiou T, Christopher Redman WG, Georgieva A. Deep Learning for Continuous Electronic Fetal Monitoring in Labor. In: 2018 40th Annual International Conference of the IEEE Engineering in Medicine and Biology Society (EMBC) [Internet]. Honolulu, HI: IEEE; 2018 [cité 5 juin 2020]. p. 5866‑9. Disponible sur: https://ieeexplore.ieee.org/document/8513625/

67. Pinsky MR, Dubrawski A. Gleaning Knowledge from Data in the Intensive Care Unit. Am J Respir Crit Care Med. 15 sept 2014;190(6):606‑10.

68. Pottenger BC, Davis RO, Miller J, Allen L, Sawyer M, Pronovost PJ. Comprehensive Unit-based Safety Program (CUSP) to Improve Patient Experience: How a Hospital Enhanced Care Transitions and Discharge Processes. Quality Management in Health Care. 2016;25(4):197‑202.

69. Raita Y. Emergency department triage prediction of clinical outcomes using machine learning models. 2019;13.

70. Rajkomar A, Oren E, Chen K, Dai AM, Hajaj N, Hardt M, et al. Scalable and accurate deep learning with electronic health records. npj Digital Med. déc 2018;1(1):18.

71. Ramkumar PN, Haeberle HS, Bloomfield MR, Schaffer JL, Kamath AF, Patterson BM, et al. Artificial Intelligence and Arthroplasty at a Single Institution: Real-World Applications of Machine Learning to Big Data, Value-Based Care, Mobile Health, and Remote Patient Monitoring. The Journal of Arthroplasty. oct 2019;34(10):2204‑9.

72. Ramkumar PN, Navarro SM, Haeberle HS, Karnuta JM, Mont MA, Iannotti JP, et al. Development and Validation of a Machine Learning Algorithm After Primary Total Hip Arthroplasty: Applications to Length of Stay and Payment Models. The Journal of Arthroplasty. avr 2019;34(4):632‑7.

73. Ratliff JK, Balise R, Veeravagu A, Cole TS, Cheng I, Olshen RA, et al. Predicting Occurrence of Spine Surgery Complications Using “Big Data” Modeling of an Administrative Claims Database: The Journal of Bone and Joint Surgery. mai 2016;98(10):824‑34.

74. Ratwani RM, Fong A. ‘Connecting the dots’: leveraging visual analytics to make sense of patient safety event reports. Journal of the American Medical Informatics Association. 1 mars 2015;22(2):312‑7.

75. Raza SA, Thalib L, Al Suwaidi J, Sulaiman K, Almahmeed W, Amin H, et al. Identifying mortality risk factors amongst acute coronary syndrome patients admitted to Arabian Gulf hospitals using machine‐learning methods. Expert Systems [Internet]. août 2019 [cité 8 déc 2020];36(4). Disponible sur: https://onlinelibrary.wiley.com/doi/abs/10.1111/exsy.12413

76. Robinson MM, Stone G, Tokarz S, Wortham B. Utilizing Actionable Data Analytics to Support Patient Navigation Enrollment and Retention Within Federally Qualified Health Centers: Journal of Public Health Management and Practice. 2017;23:S54‑8.

77. Rocchio BJ. Achieving Cost Reduction Through Data Analytics. 2016;104(4):6.

78. Ruiz-Cordell KD, Joubin K, Haimowitz S. Applying Advanced Analytical Approaches to Characterize the Impact of Specific Clinical Gaps and Profiles on the Management of Rheumatoid Arthritis: Journal of Continuing Education in the Health Professions. 2016;36(4):235‑9.

79. Ruminski CM, Clark MT, Lake DE, Kitzmiller RR, Keim-Malpass J, Robertson MP, et al. Impact of predictive analytics based on continuous cardiorespiratory monitoring in a surgical and trauma intensive care unit. J Clin Monit Comput. août 2019;33(4):703‑11.

80. Ryan J, Hendler J, Bennett KP. Understanding Emergency Department 72-Hour Revisits Among Medicaid Patients Using Electronic Healthcare Records. Big Data. déc 2015;3(4):238‑48.

81. Sandquist M, Tegtmeyer K. No more pediatric code blues on the floor: evolution of pediatric rapid response teams and situational awareness plans. Transl Pediatr. oct 2018;7(4):291‑8.

82. Schuetz G, Larson J. How to Grow Your Workforce Through Staff Optimization. Nurse Leader. août 2019;17(4):344‑6.

83. Shi L, Sun J, Yang Y, Ling T, Wang M, Gu Y, et al. Three-Dimensional Visual Patient Based on Electronic Medical Diagnostic Records. IEEE J Biomed Health Inform. janv 2018;22(1):161‑72.

84. Silahtaroğlu G, Yılmaztürk N. Data analysis in health and big data: A machine learning medical diagnosis model based on patients’ complaints. Communications in Statistics - Theory and Methods. 3 juin 2019;1‑10.

85. Singh H. iNICU – Integrated Neonatal Care Unit: Capturing Neonatal Journey in an Intelligent Data Way. J Med Syst. 2017;12.

86. Song Q, Zheng Y-J, Huang Y-J, Xu Z-G, Sheng W-G, Yang J. Emergency Drug Procurement Planning Based on Big-Data Driven Morbidity Prediction. IEEE Trans Ind Inf. déc 2019;15(12):6379‑88.

87. Spangenberg N, Wilke M, Franczyk B. A Big Data architecture for intra-surgical remaining time predictions. Procedia Computer Science. 2017;113:310‑7.

88. Stadler JG, Donlon K, Siewert JD, Franken T, Lewis NE. Improving the Efficiency and Ease of Healthcare Analysis Through Use of Data Visualization Dashboards. Big Data. juin 2016;4(2):129‑35.

89. Taylor RA, Pare JR, Venkatesh AK, Mowa H, Melnick ER, Fleischman W, et al. Prediction of In-hospital Mortality in Emergency Department Patients With Sepsis: A Local Big Data–Driven, Machine Learning Approach. ACADEMIC EMERGENCY MEDICINE. 2016;23(3):10.

90. Yang P-S, Liu C-P, Hsu Y-C, Chen C-F, Lee C-C, Cheng S-P. A Novel Prediction Model for Bloodstream Infections in Hepatobiliary–Pancreatic Surgery Patients. World J Surg. mai 2019;43(5):1294‑302.

91. Ye Y. A hybrid IT framework for identifying high-quality physicians using big data analytics. International Journal of Information Management. 2019;11.

92. Zhang W, Wang B-Y, Du X-Y, Fang W-W, Wu H, Wang L, et al. Big-data analysis: A clinical pathway on endoscopic retrograde cholangiopancreatography for common bile duct stones. WJG. 28 févr 2019;25(8):1002‑11.

93. Zhu K, Lou Z, Zhou J, Ballester N, Kong N, Parikh P. Predicting 30-day Hospital Readmission with Publicly Available Administrative Database: A Conditional Logistic Regression Modeling Approach. Methods Inf Med. 2015;54(06):560‑7.

94. Zolbanin HM, Delen D. Processing Electronic Medical Records to Improve Predictive Analytics Outcomes for Hospital Readmissions. :38.

**Additional References**

[118] C. Baechle and A. Agarwal, “A framework for the estimation and reduction of hospital readmission penalties using predictive analytics,” J Big Data, vol. 4, no. 1, p. 37, Dec. 2017, doi: 10.1186/s40537-017-0098-z.

[119] R. J. Clifford et al., “Semi-Automated Visualization and ANalysis of Trends: A ‘SAVANT’ for Facilitating Antimicrobial Stewardship Using Antistaphylococcal Resistance and Consumption as a Prototype,” Open Forum Infectious Diseases, vol. 5, no. 4, Apr. 2018, doi: 10.1093/ofid/ofy066.

[120] O. K. Danner, “Physiologically-based, predictive analytics using the heart-rate-to-systolic-ratio significantly improves the timeliness and accuracy of sepsis prediction compared to SIRS,” p. 5, 2017.

[121] C. Elvira, A. Ochoa, J. C. Gonzalvez, and F. Mochon, “Machine-Learning-Based No Show Prediction in Outpatient Visits,” IJIMAI, vol. 4, no. 7, p. 29, 2018, doi: 10.9781/ijimai.2017.03.004.

[122] G. Ficheur, L. F. Careira, R. Beuscart, and E. Chazard, “EpiHosp: A web-based visualization tool enabling the exploratory analysis of complications of implantable medical devices from a nationwide hospital database,” p. 5.

[123] F. Gonçalves, R. Pereira, J. C. Ferreira, J. B. Vasconcelos, F. Melo, and I. Velez, “Emergency Waiting Times Data Analysis,” p. 6, 2018.

[124] M. Khalifa, “Reducing Emergency Department Crowding Using Health Analytics Methods: Designing AnEvidence Based Decision Algorithm,” Procedia Computer Science, vol. 63, pp. 409–416, 2015, doi: 10.1016/j.procs.2015.08.361.

[125] M. Khalifa and I. Zabani, “Utilizing health analytics in improving the performance of healthcare services: A case study on a tertiary care hospital,” Journal of Infection and Public Health, vol. 9, no. 6, pp. 757–765, Nov. 2016, doi: 10.1016/j.jiph.2016.08.016.

[126] L. Li et al., “Sensitivity of Administrative Coding in Identifying Inpatient Acute Strokes Complicating Procedures or Other Diseases in UK Hospitals,” JAHA, vol. 8, no. 14, Jul. 2019, doi: 10.1161/JAHA.119.012995.

[127] M. K. Lodhi et al., “Predictive Modeling for End-of-Life Pain Outcome Using Electronic Health Records,” in Advances in Data Mining: Applications and Theoretical Aspects, vol. 9165, P. Perner, Ed. Cham: Springer International Publishing, 2015, pp. 56–68. doi: 10.1007/978-3-319-20910-4_5.

[128] P. N. Ramkumar et al., “Artificial Intelligence and Arthroplasty at a Single Institution: Real-World Applications of Machine Learning to Big Data, Value-Based Care, Mobile Health, and Remote Patient Monitoring,” The Journal of Arthroplasty, vol. 34, no. 10, pp. 2204–2209, Oct. 2019, doi: 10.1016/j.arth.2019.06.018.

[129] P. N. Ramkumar et al., “Development and Validation of a Machine Learning Algorithm After Primary Total Hip Arthroplasty: Applications to Length of Stay and Payment Models,” The Journal of Arthroplasty, vol. 34, no. 4, pp. 632–637, Apr. 2019, doi: 10.1016/j.arth.2018.12.030.

[130] R. M. Ratwani and A. Fong, “‘Connecting the dots’: leveraging visual analytics to make sense of patient safety event reports,” Journal of the American Medical Informatics Association, vol. 22, no. 2, pp. 312–317, Mar. 2015, doi: 10.1136/amiajnl-2014-002963.

[131] S. A. Raza et al., “Identifying mortality risk factors amongst acute coronary syndrome patients admitted to Arabian Gulf hospitals using machine‐learning methods,” Expert Systems, vol. 36, no. 4, Aug. 2019, doi: 10.1111/exsy.12413.

[132] K. D. Ruiz-Cordell, K. Joubin, and S. Haimowitz, “Applying Advanced Analytical Approaches to Characterize the Impact of Specific Clinical Gaps and Profiles on the Management of Rheumatoid Arthritis:,” Journal of Continuing Education in the Health Professions, vol. 36, no. 4, pp. 235–239, 2016, doi: 10.1097/CEH.0000000000000119.

[133] J. Ryan, J. Hendler, and K. P. Bennett, “Understanding Emergency Department 72-Hour Revisits Among Medicaid Patients Using Electronic Healthcare Records,” Big Data, vol. 3, no. 4, pp. 238–248, Dec. 2015, doi: 10.1089/big.2015.0038.

[134] Q. Song, Y.-J. Zheng, Y.-J. Huang, Z.-G. Xu, W.-G. Sheng, and J. Yang, “Emergency Drug Procurement Planning Based on Big-Data Driven Morbidity Prediction,” IEEE Trans. Ind. Inf., vol. 15, no. 12, pp. 6379–6388, Dec. 2019, doi: 10.1109/TII.2018.2870879.
